# sRNAscanner: A Computational Tool for Intergenic Small RNA Detection in Bacterial Genomes

**DOI:** 10.1371/journal.pone.0011970

**Published:** 2010-08-05

**Authors:** Jayavel Sridhar, Suryanarayanan Ramkumar Narmada, Radhakrishnan Sabarinathan, Hong-Yu Ou, Zixin Deng, Kanagaraj Sekar, Ziauddin Ahamed Rafi, Kumar Rajakumar

**Affiliations:** 1 Centre of Excellence in Bioinformatics, School of Biotechnology, Madurai Kamaraj University, Madurai, Tamilnadu, India; 2 Bioinformatics Centre, Indian Institute of Science, Bangalore, Karnataka, India; 3 Department of Infection, Immunity and Inflammation, University of Leicester, Leicester, United Kingdom; 4 Department of Clinical Microbiology, University Hospitals of Leicester NHS Trust, Leicester, United Kingdom; 5 Laboratory of Microbial Metabolism and School of Life Sciences and Biotechnology, Shanghai Jiaotong University, Shanghai, People's Republic of China; Cairo University, Egypt

## Abstract

**Background:**

Bacterial non-coding small RNAs (sRNAs) have attracted considerable attention due to their ubiquitous nature and contribution to numerous cellular processes including survival, adaptation and pathogenesis. Existing computational approaches for identifying bacterial sRNAs demonstrate varying levels of success and there remains considerable room for improvement.

**Methodology/Principal Findings:**

Here we have proposed a transcriptional signal-based computational method to identify intergenic sRNA transcriptional units (TUs) in completely sequenced bacterial genomes. Our sRNAscanner tool uses position weight matrices derived from experimentally defined *E. coli* K-12 MG1655 sRNA promoter and rho-independent terminator signals to identify intergenic sRNA TUs through sliding window based genome scans. Analysis of genomes representative of twelve species suggested that sRNAscanner demonstrated equivalent sensitivity to sRNAPredict2, the best performing bioinformatics tool available presently. However, each algorithm yielded substantial numbers of known and uncharacterized hits that were unique to one or the other tool only. sRNAscanner identified 118 novel putative intergenic sRNA genes in *Salmonella enterica* Typhimurium LT2, none of which were flagged by sRNAPredict2. Candidate sRNA locations were compared with available deep sequencing libraries derived from Hfq-co-immunoprecipitated RNA purified from a second Typhimurium strain (Sittka et al. (2008) PLoS Genetics 4: e1000163). Sixteen potential novel sRNAs computationally predicted and detected in deep sequencing libraries were selected for experimental validation by Northern analysis using total RNA isolated from bacteria grown under eleven different growth conditions. RNA bands of expected sizes were detected in Northern blots for six of the examined candidates. Furthermore, the 5′-ends of these six Northern-supported sRNA candidates were successfully mapped using 5′-RACE analysis.

**Conclusions/Significance:**

We have developed, computationally examined and experimentally validated the sRNAscanner algorithm. Data derived from this study has successfully identified six novel *S.* Typhimurium sRNA genes. In addition, the computational specificity analysis we have undertaken suggests that ∼40% of sRNAscanner hits with high cumulative sum of scores represent genuine, undiscovered sRNA genes. Collectively, these data strongly support the utility of sRNAscanner and offer a glimpse of its potential to reveal large numbers of sRNA genes that have to date defied identification. sRNAscanner is available from: http://bicmku.in:8081/sRNAscanner or http://cluster.physics.iisc.ernet.in/sRNAscanner/.

## Introduction

Systematic experimental and computational approaches have led to the identification of ∼92 small RNAs (sRNAs) in *Escherichia coli* K12 MG1655 alone [1]. Many sRNAs have been assigned regulatory roles in the survival and physiology of the organism [2]. Prokaryotic sRNAs are known to play roles in regulation of sporulation [3], sugar metabolism [4], iron homeostasis [5], survival under oxidative stress [6], DNA damage repair, maintenance of cell surface components [7] and regulation of pathogenicity [8]. Though sRNAs do not code for peptides they exert their function through antisense modes by RNA–RNA base pairing [9], [10] or by antagonizing target proteins through RNA–protein interactions [11]. Genomic screens for sRNAs have been most extensively conducted in the model organisms *E. coli* K-12 [12], [13] and *Bacillus subtilis* [3]. More recently, significant numbers of sRNAs in pathogens such as *Staphylococcus aureus* [14], *Pseudomonas aeruginosa* [15] and *Listeria monocytogenes* [16] have been identified, though functional roles of the majority remain to be determined.

Most computational methods, such as QRNA [17] and Intergenic Sequence Inspector [18], use intergenic sequence conservation among related genomes to identify sRNAs. By contrast, the RNAz [19] and sRNAPredict [15], [20] programs utilize estimated thermodynamic stability of conserved RNA structures and existing ‘orphan’ promoter and terminator annotations for sRNA predictions, respectively. Previous studies by Argaman et al. [12], Chen et al. [21], Pfeiffer et al. [22] and Valverde et al. [23] had used promoter and terminator signals to predict sRNAs but did not provide computational scripts for general use. This study implements a generic transcriptional signal detection strategy and applies it systematically to obtain reproducible computational results and matching ‘prediction scores’. Furthermore, sRNAPredict [15], [20] and SIPHT [24] require available promoter information and databases of rho-independent terminators predicted by TransTermHP [25] to identify sRNAs. Moreover, sRNAPredict2 requires as inputs sequence and structure conservation data as identified by Blast and QRNA, respectively, markedly hampering detection of sRNAs mapping to non-conserved intergenic sequences. The proposed tool overcomes these limitations by searching genome sequences for orphan transcriptional signals and integrating signal co-ordinates to identify candidate intergenic sRNAs without any pre-requirements.

Comparative genomic approaches are restricted to identifying sRNA candidates located within conserved genomic backbone regions common to closely related bacteria [26]. However, most bacterial species have significant cumulative spans of multiple strain-specific sequences or islands, dispersed along the genome, many of which play key adaptive and/or pathogenesis-related roles [27], [28]. Indeed, genomic island-borne sRNAs have been identified in *S. aureus* [14] and *Salmonella enterica* serovar Typhimurium [22], [29]. Furthermore, sRNAs transcribed from strain-specific regions of *S.* Typhimurium were reported to partake in complex networks for stress adaptation and virulence regulation [8], [22], [28], [29] leading Toledo-Arana et al. [8] to emphasize the need for identification of strain-specific sRNAs in pathogens. *S.* Typhimurium is an important food-borne pathogen that causes a substantial burden of diarrhoeal disease globally. Life-threatening systemic infections can also occur in those with severe co-morbidities, at extremes of age and/or with impaired immune systems.

We have constructed a position weight matrix (PWM) based tool named sRNAscanner, using *E. coli* K-12 MG1655 sRNA-specific transcriptional signals as positive training data, for the identification of intergenic sRNAs. Experimentally characterized *E. coli* sRNA promoters appear to vary slightly in base distribution frequencies when compared to *E. coli* mRNA promoters (Table S1a), though it remains possible that observed differences may be statistically insignificant. sRNAscanner cut-off thresholds were identified using the known *E. coli* K-12 MG1655 sRNAs as a positive dataset [30]. The predictive abilities of sRNAscanner and sRNAPredict2 [20] were then compared by analysing 13 bacterial genomes representative of diverse species. As a specific case study, we analyzed a *S.* Typhimurium complete genome sequence and experimentally validated a small set of previously uncharacterized predictions. Our results strongly support the accuracy and utility of sRNAscanner as a tool for the discovery of novel sRNA genes within intergenic regions of bacterial genomes and hint at the broader power of customized PWMs as a generic strategy for detection of defined genomic features in diverse bacterial genomes.

## Methods

### Summary of the sRNAscanner program

**Figure 1:**
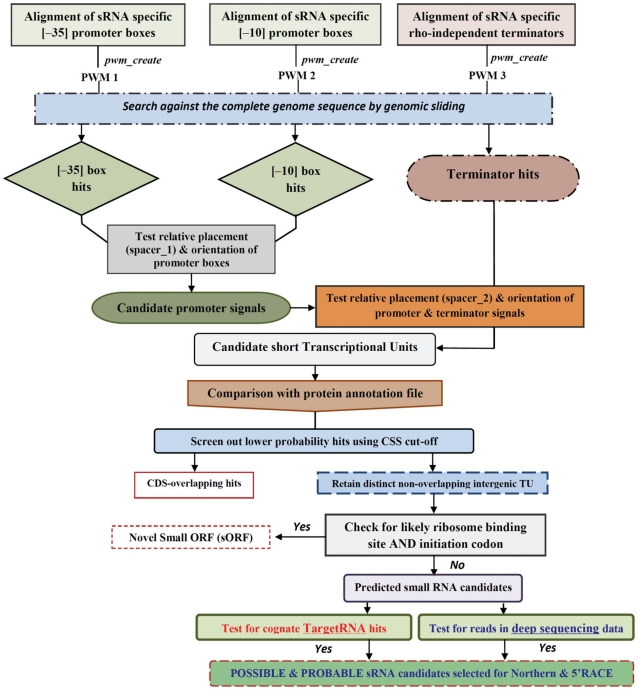
Flowchart illustrating an overview of the sRNAscanner algorithm. The final step was performed using the web-based TargetRNA [41] utility and/or by comparison of sRNAscanner hits with RNA deep sequencing datasets. The output dataset obtained is shown as the red outlined box at the bottom of the figure. sRNAscanner hits supported by TargetRNA only are classed as possible sRNA candidates, whilst those supported by deep seqeuncing are considered as probable sRNA candidates. Details of parameter values used in this study are as indicated in the text.

sRNAscanner uses as inputs matching complete bacterial genome sequence and protein coding table files in standard FASTA and tab-delimited text formats, respectively, to identify sRNA genes in intergenic regions. The sRNAscanner suite consists of algorithms to perform the following functions: (a) construct PWMs from sRNA-specific transcriptional signals, (b) search complete genome sequences using constructed PWMs to identify ‘orphan’ intergenic promoter and terminator locations, (c) perform coordinate based integration of promoter/terminator signals to define putative intergenic transcriptional units (TU) and (d) select predicted TUs based on **c**umulative **s**um of **s**cores (CSS) values above a nominated threshold. The CSS value is determined by summating three individual matrix-specific **s**um of **s**cores (SS) values for each candidate TU (see below for calculation of SS value). sRNAscanner uses pre-computed PWM and the following pre-defined parameters to predict intergenic sRNAs: promoter box 1 SS value (≥2), promoter box 2 SS value (≥2), terminator SS value (≥3), spacer 1 range (defines distance between promoter boxes 1 and 2; 12–18), spacer 2 range (defines distance between promoter box 2 and terminator signal; 40–350), Unique Hit value (200) and CSS (≥14). The Unique Hit value identifies potential TU from a set of overlapping hits based on the presence of closely located start coordinates mapped within a defined window size which by default is set at 200 bp. sRNAscanner selects the TU with the maximum CSS value from each overlapping set as a unique representative hit for the set. Note: all parameters can be altered by users as required. Predicted TUs are examined for the presence of a putative ribosome binding site and initiation codon; if both signals are identified the TUs are classified as coding for putative mini-proteins [28]. Remaining TUs are considered to code for candidate sRNA molecules. A flowchart summarizing the sRNAscanner algorithm is shown in Figure 1.

### Construction of PWMs from training data

sRNAscanner computes a PWM of four rows and *x* columns for *N* input sequences each having *x* residues; *N* and *x* can be any positive integer. The program uses multiple sequences of sRNA-specific transcriptional signals in fasta format as input for the construction of alignment matrices. The alignment matrix captures the number of occurrences, *n_i,j_*, of letter *i* at position *j* across the set of aligned sequences. Subsequently, actual occurrence values were converted into log-odd scores; values that reflect the positional weights of each of the four bases (A, T, G, C) at each position. Frequency calculations and scoring schemes were adopted from previous algorithms and the positional weights were derived from the alignment matrix itself. A PWM was then derived from the above alignment matrix using the following formula (see Hertz and Stormo, 1999 [31] for details):
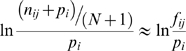
In this formula *N* is the total number of input sequences and *p_i_* is the *a priori* probability of the letter *i* occurring at position *j* of an input sequence; by definition for a four component system (A, C, G & T) this expected frequency is 0.25 for each of the four nucleotides, *f_i,j_* = *n_i,j_*/N is the frequency of the letter *i* in position *j*. Importantly, the precise genomic base frequency of the training or test genomes do not have a role in the construction of PWM. The log-odd scores are used for the construction of PWM; the algorithm was implemented using the PWM_create module of the sRNAscanner program. We have used ten promoter boxes and twenty one rho-independent terminators [21] of experimentally-verified *E. coli* K-12 sRNA genes as training data to construct PWM1 (promoter box1), PWM2 (promoter box2) and PWM3 (rho-independent terminator) (Table S1 and Figure S1).

### Identification of intergenic sRNA specific transcriptional units

PWM1, PWM2 and PWM3 matrices were used individually to scan entire genome sequences, one nucleotide at a time, by a sliding window method as described previously [31]. The width of each sliding window was equal to the length of its matching input PWM. The matrix-specific SS value of each DNA sequence window was calculated by adding the PWM-determined scores corresponding to each of the respective bases within the window as described previously [31]. Each successive sliding window was assigned a SS value and it was compared against a selected threshold SS value obtained by analysis of the 92 known *E. coli* K-12 sRNA genes from the sRNAMap and Rfam datasets (http://srnamap.mbc.nctu.edu.tw/). sRNAscanner was run with an arbitrary minimum SS value of 1 for each of the three matrices to identify potential intergenic TUs which were then compared manually with the known K-12 sRNA genes to identify concordant pairs. Using these criteria and no imposed CSS cut-off, 66 of the 92 known sRNAs were identified as possessing sRNAscanner-detectable potential transcriptional signals (Table S2). Re-iterative empirical analyses using progressively higher matrix-specific SS values were performed to identify matrix-specific default SS thresholds that sought to maximize sensitivity whilst minimizing false-positive hits; SS cut-offs determined were as mentioned previously. Sequences having PWM1-, PWM2- and PWM3-specific SS values above the threshold scores were selected as potential promoter box 1, promoter box 2 and terminator signal hits, respectively. Next, the orientation, relative position and spacing of PWM-detected hits were examined against pre-defined allowable ranges for spacer 1 and spacer 2 to identify potential TUs. Spacer parameters used were based on analysis of the length and transcriptional signal spacing features of known *E. coli* and other *Enterobacteriaceae* sRNAs. Sequences satisfying both spacer checks and a selected CSS cut-off value were identified as likely TUs. The PWM3 SS value was expected to contribute most to the CSS score as for the known *E. coli* K-12 TUs detected by the program, PWM3 scores varied from 4.54–11.19, whilst the top values for PWM1 and PWM2 were 4.98 and 6.03, respectively. Importantly, higher SS values on one or both of the other matrices would not have compensated for a single below-threshold score. Identified TUs were compared with protein coding annotation files. Non-redundant, intact, non-overlapping TUs identified within intergenic regions alone and lacking putative ribosome binding sites and start codons were reported as probable sRNA-specific intergenic TUs.

### sRNAscanner availabitlity and requirements

Project name: sRNAscanner; Home page: http://bicmku.in:8081/sRNAscanner or http://cluster.physics.iisc.ernet.in/sRNAscanner/; Operating system: Linux/Unix platforms; Programming language: C++; Compiler: g++/gcc 4.2 or higher; License: GNU GPL.

### Bacterial strain and growth conditions

*S. enterica* Typhimurium wild type strain SL1344 (JVS-1574, MPIIB culture collection) was used for experimental validation. For early stationary phase (ESP) and late stationary phase (LSP) cultures, 25 ml of Luria-Bertani broth was inoculated with a 1/100 overnight culture and grown at 37°C in a shaking incubator (220 rpm) in a 100 ml flask. Optical density at 600 nm (OD_600_) was monitored. Two ESP cultures (OD_600_ = 0.5 [OD-0.5], OD_600_ = 2.0 [OD-2.0]) and four LSP cultures (3 h [3H], 6 h [6H], 9 h [9H] and overnight [ON] post-OD_600_ = 2.0) were obtained. Approximately 10^8^ ESP (OD_600_ = 0.5) cells were treated with mitomycin C (0.5 µg/ml) [SOS], acidic LB (pH 5.4) [Acid] or cold shock (15°C) [Cold] for 30 min to induce an SOS response, acid stress or cold shock conditions, respectively. Abbreviations shown are to describe the eleven growth conditions. *Salmonella* pathogenicity island 1 (SPI-1) induced cultures [SPI-1] were grown with high salt-containing LB broth (0.3 M NaCl) for 12 hours at 37°C/220 rpm in tightly closed tubes. *Salmonella* pathogenicity island 2 (SPI-2) induced cultures [SPI-2] were prepared by inoculating 70 ml of SPI-2 medium [32] in 250 ml flasks, with 1/100 inoculums grown in SPI-2 medium overnight, and incubated at 37°C/220 RPM until reaching an OD_600_ = 0.3. The above cultures were spun down and the cell pellets mixed with stop mixture [95% ethanol (v/v), 5% phenol (v/v)] and immediately frozen in liquid nitrogen.

### RNA isolation and Northern blot analysis

Total RNA was prepared from frozen cells using the TRizol (Invitrogen) method and treated with DNase I (Fermentas) as described previously [32]. Approximately 10 µg of RNA for each growth condition was added to 2× RPA buffer and run on 6% polyacrylamide/7 M urea gels, along with a pUC8 DNA ladder (Fermentas). After separation RNA was transferred to Hybond-XL nylon membranes (GE Healthcare) and UV cross-linked. Potential sRNA transcripts were detected using γ-ATP end-labeled oligonucleotide probes (Table S3).

### 5′ RACE mapping of RNA transcripts

5′RACE experiments were performed as described by Vogel and Wagner [33]. In summary, primary transcripts were treated with tobacco acid pyrophosphatase (TAP), ligated to A4 RNA adapters (500 pmol) at the 5′ends and reverse transcribed into cDNA with random hexamers (400 ng) using Superscript II Reverse Transcriptase (Invitrogen). Next, the first strand of the cDNA molecule was PCR amplified using an adapter-specific primer (JVO-0367) and matching sRNA-specific primer (Table S3). Amplified 5′ RACE products were cloned into TOPO pCR2.1 and sequenced from both ends with M13 primers.

## Results and Discussion

### Optimization of sRNAscanner with known *E. coli* K-12 MG1655 (NC_000913) sRNA data

**Figure 2:**
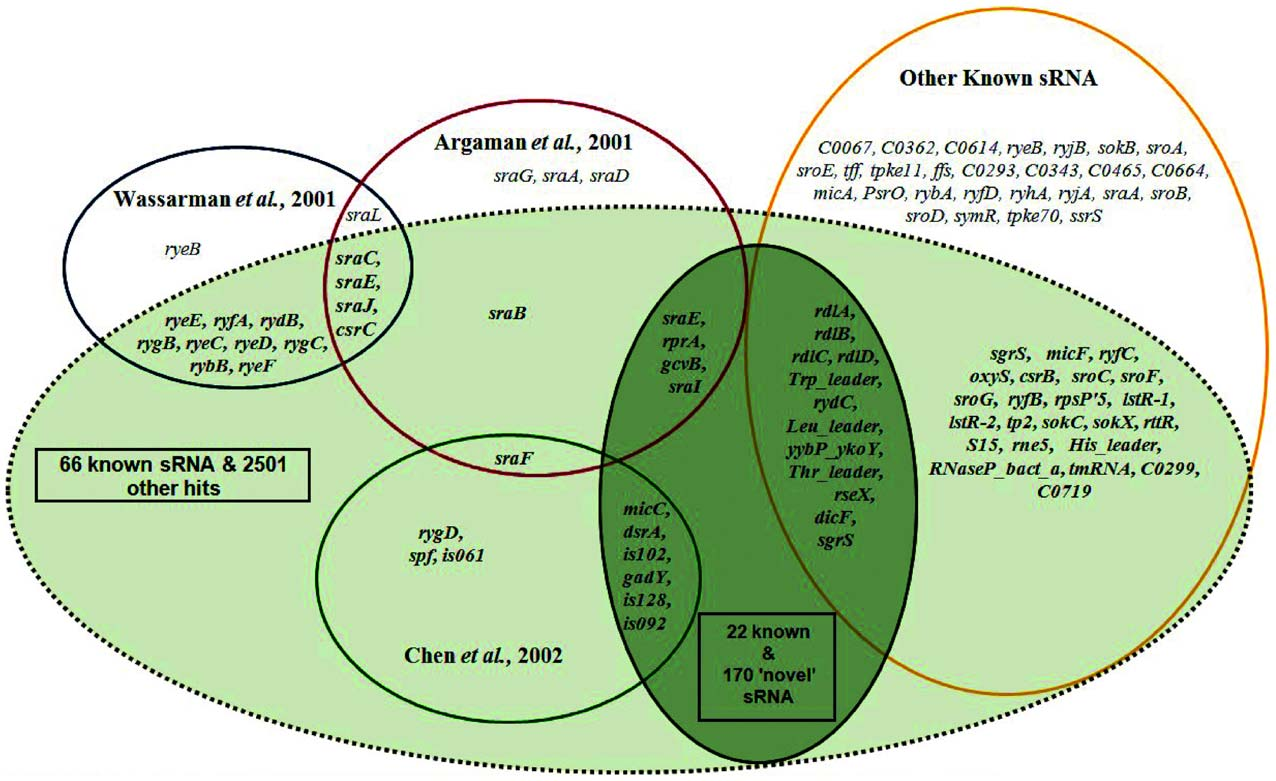
Venn diagram showing the set of known *E. coli* K-12 MG1655 sRNA genes detected or missed by sRNAscanner. The program was run using the training set-derived PWMs and parameters described in the text. The pale green elipse shown in dotted outline highlights the set of 66 known sRNA genes detected when the program was run without a CSS cut-off threshold. The darker green vertical oval indicates the set of 22 known sRNAs and a further 170 potentially novel intergenic sRNA detected using a CSS>14 cut-off. The sets of known *E. coli* K-12 MG1655 sRNA genes predicted bioinformatically by Wassarman et al. [13], Argaman et al. [12] and Chen et al. [21] are shown in blue-, red- and green-outline ovals, respectively. A further 61 sRNA genes identified through diverse experimental and bioinformatic means are shown in the yellow-outline oval.

**Figure 3:**
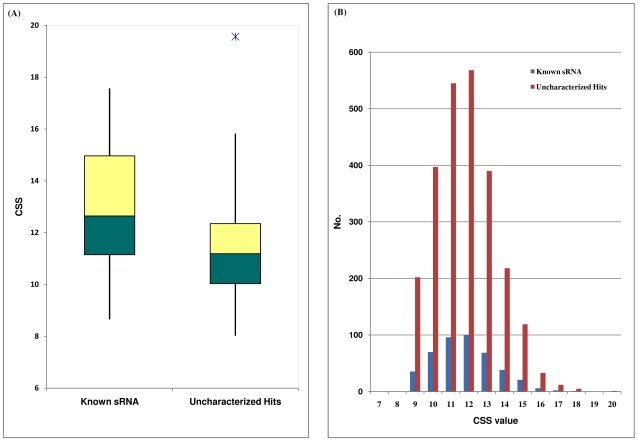
Distribution of sRNAscanner cumulative sum of scores (CSS) for known sRNA and uncharacterized hits in *E. coli* K-12 MG1655. The program was run using default parameters mentioned in the text. (**A**) The lower and top boundaries of the whisker plot boxes represent the 25^th^ and 75^th^ quartiles, respectively. The vertical lines extending from the boxes indicate the full range of the remaining CSS values with the exception of a single outlier, indicated as a cross, for the uncharacterized hits plot. (**B**) Histogram showing the CSS distributions of the two sets of sRNAscanner hits.

We analysed the *E. coli* K-12 MG1655 (NC_000913) genome using pre-defined parameters (see User Guide) and matrices trained with data from ten promoter boxes and twenty one rho-independent terminators [21] of experimentally verified *E. coli* K-12 sRNA genes. To maximize sensitivity at the expense of specificity, we ran this analysis without application of a CSS cutoff. Predicted intergenic sRNA-specific transcriptional units were compared with the 92 reported *E. coli* K-12 sRNAs available in sRNAmap [1] and/or Rfam [34]. Physical locations of 66 of the 92 experimentally-validated sRNAs fully or partially overlapped with sRNAscanner-identified putative TUs. However, application of the program without a CSS cut-off led to extremely low specificity with >2,500 putative intergenic TU identified. Subsets of known MG1655 sRNA predicted by sRNAscanner and other computational and experimental methods are shown as a Venn diagram (Figure 2). The mean and standard deviation of the CSS of experimentally verified MG1655 sRNA transcriptional units detected by sRNAscanner were used to define a stringent CSS cut-off value of 14 (mean + standard deviation = 13.87). Nevertheless, the substantial overlap between whisker plots of CSS values for the known sRNAs and the uncharacterized sRNAscanner hits (Figure 3A) and the fact that these two sets remained unresolved even when CSS score distributions were plotted as a histogram (Figure 3B), suggested that many genuine *E. coli* K-12 intergenic TUs remained to be experimentally defined or that the matrices and/or the sRNAscanner algorithm lacked specificity. Interestingly, the single uncharacterized hit outlier with a CSS = 19.56 has also been predicted by SIPHT (Figure 3A). Lists of sRNAscanner-predicted (CSS>14) known and novel candidate sRNA TUs in MG1655 are as shown (Table S2 and Table S4).

### Analysis of sRNAscanner performance characteristics

**Figure 4:**
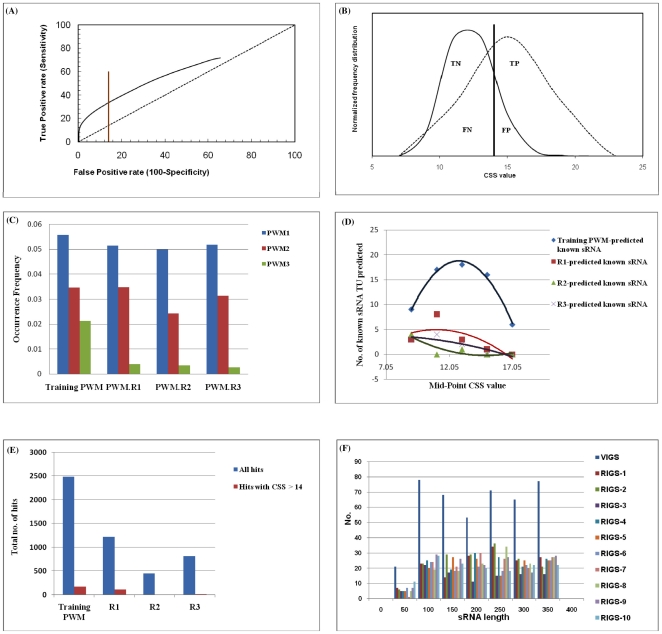
The three approaches used to estimate the specificity of sRNAscanner. Conventional ROC (**A**) and normalized frequency distribution (**B**) plots were generated following analysis of the *E. coli* K-12 genome. The brown line in (A) denotes the point on the ROC curve which corresponds to CSS = 14. For these analyses, the set of 92 known sRNA were defined as the true positive set. Random matrices-based specificity analysis data are shown in panels (C), (D) and (E). (**C**) Histogram indicating the occurrence frequencies or predictions per nucleotide of intergenic hits with each of the three training set-derived matrices and the matching R1, R2 and R3 randomly shuffled versions of these matrices. The test genome sequence analysed was that of *E. coli* K-12 MG1655. (**D**) Graph showing the numbers of known MG1655 sRNA TU predicted by sRNAscanner within each of five CSS ranges plotted against the mid-point CSS value for the CSS range when the program was run with the training set-derived PWM or each of the three matching sets of random PWM in turn. (**E**) Bar graph showing the total numbers of hits (known and uncharacterized) predicted by sRNAscanner when the program was run with the training set-derived PWM and each of the matching random PWM. (**F**) Histogram showing the distribution of candidate ‘sRNA TUs’ predicted by length of sRNA within a composite sequence comprising concatenated intergenic sequences from *E. coli* K-12 (VIGS) and ten randomly suffled variants on this sequence (RIGS-1 – RIGS-10).

sRNAscanner was run with the training set derived matrices and pre-defined parameters. Excluding the 10 sRNAs used to inform the PWM1 and PWM2 matrices, sRNAscanner (CSS>14) detected 24% of the known *E. coli* K-12 sRNA genes [1]. Assessment of the specificity of sRNA prediction tools remains extremely challenging as there are no gold standards and known bacterial sRNAs are likely to represent no more than the tip of a vast ‘RNome’ iceberg. Even experimental validation is problematic as individual sRNA may only be expressed under highly specific conditions and/or at extremely low levels. We have attempted to examine the specificity of sRNAscanner through three bioinformatics approaches. sRNA genes used to inform the training dataset were included in these subsequent analyses. Firstly, we have generated a conventional Receiver Operating Characteristic (ROC) plot [35] based on analysis of the *E. coli* K-12 genome (Figure 4A). The set of known K-12 sRNAs predicted by sRNAscanner were defined as the ‘True positive’ set and the impact of the full range of CSS cut-off values was assessed. The ROC plot and related normalized frequency distribution graph (Figure 4B) suggested a major sensitivity–specificity sacrifice with there being no classical optimum point; favoring either led to a marked deterioration of the other. However, even by these criteria the sensitivity (*Sn*) – specificity (*Sp*) performance of sRNAscanner at CSS>14 (*Sn* = 32%; *Sp* = 95%) was comparable to that of sRNAPredict2 (*Sn* = 20%; *Sp* = 96%). Secondly, we compared the performance of the pre-computed training-set-derived PWMs with those of randomly generated ‘equivalent’ matrices and used both sets of matrices to analyse the *E. coli* K-12 genome sequence. Equivalent random matrices were generated by randomly shuffling entire columns within each matrix (R1 random matrices) (Figure S2), the numbers within individual columns (R2 random matrices) (Figure S3), and a combination of these two shuffling strategies (R3 random matrices) (Figure S4). This approach preserved the precise SS characteristics for matching genuine and random matrices and allowed the same SS and CSS thresholds to be used. However, only the R1 random matrices represented the same combination of nucleotide preferences, though present in distinct permutations as compared to the original matrices. The training and random PWM sets were used to search the *E. coli* K-12 genome to identify occurrences of each motif and, through integration of these data, TU-like arrangements. The **o**ccurrence **f**requencies (OF) of individual motifs were defined as the number of predictions per nucleotide of the genome. The ratios of OF obtained with the random and rationally-derived original matrices were expected to be inversely proportional to the ratios of matrix specificities [36]. However with the exception of the comparison between the genuine and R1 versions of PWM2, all three training PWM had higher OF than matching random matrices when applied to the K-12 genome sequence (Figure 4C). This was most marked for PWM3 with its three random versions exhibiting less than 20% of the hits observed with the training set-derived matrix. These data strongly argued against the random nature of bacterial intergenic DNA and demonstrated the relative abundance of terminator-like motifs in intergenic regions. Hits identified by the random matrices were compared with known sRNA regions to identify the number of known sRNA TUs detected. The stringent requirement for the correctly ordered, orientated and appropriately spaced occurrence of each of the three independently detected transcriptional signals was expected to filter out much of the noise. Indeed, use of the training dataset-derived PWMs resulted in identification of 66 known sRNA TUs (CSS scores [mean, range]: 12.87, 8.65–17.57), while use of the R1 random PWM, the best performing of the random versions, yielded only 14 known sRNA TUs with lower CSS scores (11.42, 9.77–14.09). The R2 and R3 shuffled matrices identified 5 and 9 potential sRNA TUs, respectively. Hence, the training matrices detected more than four times as many known sRNA TUs but only approximately twice as many total ‘TU’ hits as the R1 matrices (Figures 4D and 4E). Nevertheless, as the random matrices yielded up to 68% as many total ‘TU’ hits as the training set-derived PWMs it would appear that even with a stringent CSS>14 cut-off, that at best only about 40% of positive calls were valid. As a third approach, we hypothesized that the ratio of the numbers of hits obtained with the full complement of concatenated genuine intergenic DNA to those found on randomly shuffled intergenic sequences would provide a qualitative measure of specificity. The concatenated sequence comprising all K-12 intergenic sequences fused end-to-end (VIGS) was subjected to random nucleotide shuffling to generate ten random variants (RIGS-1 – RIGS-10). A length distribution histogram of the ‘sRNA’ hits in the VIGS and RIGS sequences is shown in Figure 4F. Consistent with a moderate level of specificity, the concatenated native intergenic sequence yielded approximately three times as many hits as those identified on the ‘average’ random intergenic sequence (435 *vs* 152) (Table S5). Use of future additional filters and/or genus-adapted PWMs may lead to incremental increases in specificity, perhaps with minimal loss of sensitivity. For example, TransTermHP-2.07-predicted rho-independent terminators in *E. coli* K-12 and *S.* Typhimurium LT2 typically exhibited PWM3 scores of ≥6 as opposed to the PWM3 minimum score criterion of >3, suggesting a possible route to specificity gain.

### Head to head comparison of sRNAscanner and sRNAPredict2

A diverse group of bacterial genome sequences representative of *Enterobacteriaceae*, *Vibrionaceae*, *Pseudomonadaceae*, *Bacillaceae*, *Clostridiaceae*, *Chlamydiaceae* and *Lactobacillaceae* were analyzed using sRNAscanner. Intergenic transcriptional unit data derived from sRNAscanner analyses were compared with previously reported sRNAPredict2 results [20]. Manual curation of these predictions identified partial or complete overlaps with known sRNAs. sRNAscanner (CSS>14) and sRNAPredict2 detected a total of 180 (*Sn* = 31.3%) and 184 (*Sn* = 32%) known sRNA genes, respectively, across all 13 bacterial genomes investigated (Table 1). However, across the genomes analyzed 0 to 23 known sRNAs per genome, comprising a total of 88 known sRNAs, were predicted uniquely by sRNAscanner. By comparison, 92 known sRNAs were predicted uniquely by sRNAPredict2. However, sRNAPredict2 yielded appreciably more uncharacterized hits than sRNAscanner (2953 *vs* 2344), suggesting a higher signal-to-noise ratio for the latter. Similarly, large numbers of novel hits missed by sRNAPredict2 were predicted by sRNAscanner, and *vice versa*. Indeed, combined use of the two tools may potentially offer a degree of cross-validation. However, sRNAscanner as optimized presently appeared to be more appropriate for the analysis of genomes of *Enterobacteriaceae* and other medium/low G+C organisms. sRNAscanner sensitivity versus known sRNAs ranged from 51% for *Clostridium tetani* E88 (28.6% G+C) to 24% for *Salmonella* Typhi CT18 (51.9% G+C) to 0% for *Mycobacterium tuberculosis* CDC1551 (65.6% G+C). Detailed lists of known and putative sRNA regions predicted by sRNAscanner in the above genomes are provided as supplementary data files (see Table S4 and File S1).

**Table 1:** Comparison of sRNA gene predictions obtained using sRNAscanner and sRNAPredict2.

Bacterial strain/GenBank Acc. No/%G+C/No. of known sRNA genes^a^	sRNAscanner^b^		sRNAPredict2^b^		sRNAscanner AND sRNAPredict2^b^		Unique to sRNAscanner^b^	Unique to sRNAPredict2^b^
	Known	Novel	Known	Novel	Known	Novel	Known	Known
*Bacillus anthracis* Ames*/AE016879/35.4/**97**	49	535	60	869	34	97	15	26
*Clostridium tetani* E88*/AE015927/28.6/**53**	27	285	20	132	17	27	10	3
*Chlamydia trachomatis* * D-UW-3-Cx/AE001273/41.3/**3**	1	27	0	43	0	6	1	0
*Helicobacter pylori* 26695*/AE000511/38.9/**4**	2	107	0	50	0	4	2	0
*Mycobacterium tuberculosis* CDC1551*/AE000516/65.6/**15**	0	1	0	50	0	0	0	0
*Pseudomonas aeruginosa* PAO1*/AE004091/66.6/**26**	3	17	4	34	1	0	2	3
*Salmonella enterica* serovar Typhi CT18*/AL513382/51.9/**63**	15	175	27	572	11	31	4	16
*Staphylococcus aureus* N315*/BA000018/32.8/**32**	17	253	24	144	12	30	5	12
*Streptococcus pneumoniae* TIGR4*/AE005672/39.7/**25**	9	190	3	62	3	16	6	0
*Streptococcus pyogenes* M1 GAS*/AE004092/38.5/**16**	4	162	6	56	3	8	1	3
*Yersinia pestis* KIM/AE009952*/47.7/**42**	7	287	17	755	7	46	0	10
*Salmonella typhimurium* LT2$/AE006468/52.2/**106**	24	135	4	65	1	0	23	3
*Escherichia coli* K12-MG1655$/U00096/50.8/**92**	22	170	19	121	3	7	19	16

a Complete lists of non-coding sRNA (including *cis*-regulatory & leader RNA) for the selected genomes were obtained from the Rfam database [34] which excludes tRNA and rRNA. Additional known sRNAs collated from the sRNAMap database [1] were also included in the lists.

b Number of known and novel sRNA genes predicted using the following strategies: (1) sRNAscanner, (2) sRNAPredict2, (3) Predicted by **BOTH** sRNAscanner **AND** sRNAPredict2, (4) Predicted **UNIQUELY** by sRNAscanner and **NOT** by sRNAPredict2, (5) Predicted **UNIQUELY** by sRNAPredict2 and **NOT** by sRNAscanner. The sRNAscanner predictions were performed using the selected CSS cut-off (CSS>14).

* The sRNAPredict2 data shown for 11 genomes were reproduced from Livny *et al.*, 2006 [20].

$ K-12 and LT2 were newly analysed in this study using the latest version of sRNAPredict2 with the default parameters and blast partners described by Livny *et al.*, 2006 [20].

### Identification of novel sRNAs in *Salmonella enterica* Typhimurium SL1344

**Figure 5:**
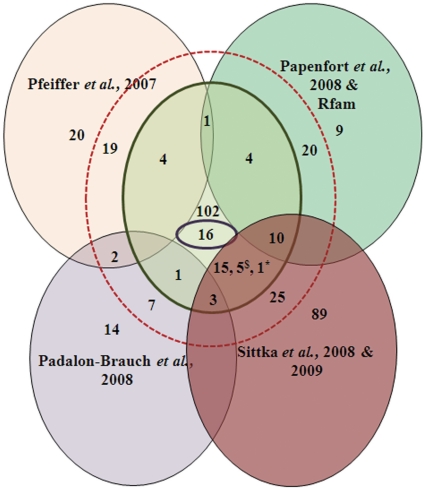
Venn diagram showing the numbers of known sRNAs in *Salmonella* Typhimurium LT2 that have been identified or reported by Pfeiffer et al. [**22**], Papenfort et al. [**39**] and Rfam [**34**], Padalon-Brauch et al. [**29**] and Sittka et al. [**32**], [38****]. The circles shown in red dotted outline and green solid outline, excluding the central pale green curve-sided triangular area, indicate the numbers of known sRNAs predicted by sRNAscanner without and with the use of a CSS cut-off (CSS>14), respectively. The central pale green curve-sided triangular area, including the innermost circle outlined in purple, represents the 118 novel, intergenic, non-overlapping candidate sRNAs predicted in this study; the innermost circle outlined in purple represents the 16-member subset comprising sRNA candidates found to have likely mRNA transcripts by comparison with RNA deep sequencing datasets [32], [38]. The $ superscript symbol indicates the five candidates belonging to both the Pfeiffer et al. [22] and Sittka et al. [32], [38] sets; the asterisk symbol denotes the one sRNA candidate mapping to the Padalon-Brauch et al. [29], Papenfort et al. [39] and Sittka et al. [32], [38] sets.

Analysis of the *S.* Typhimurium LT2 genome using sRNAscanner under default conditions yielded a total of 38 known and 118 novel candidate sRNAs (Figure 5, Table S4). The genomic locations of the 118 novel sRNA candidates were compared with putative intergenic transcripts detected in deep sequencing libraries derived from Hfq-co-immunoprecipitated RNA obtained from *S.* Typhimurium SL1344 grown under multiple conditions [32], [37], [38] [unpublished data, J. Vogel]. *S.* Typhimurium SL1344 was used for all subsequent experimental validation as no comparable RNA deep sequencing dataset was available for *S.* Typhimurium LT2. Sixteen novel sRNA candidates were detected by both sRNAscanner and deep sequencing analysis (Table 2).

**Table 2:** Thirty three novel candidate sRNAs predicted by sRNAscanner AND RNA deep sequencing data or TargetRNA identification of putative cognate targets.

sRNA Id^a^	Start^b^	End^c^	Length^c^	Flanking gene id^d^	5′RACE mapping^e^	Strand^g^	Target mRNA^h^	mRNA Function^i^	Northern^f^	Reference^l^
**sRNA1**	257730	257795	∼66	STM0219/STM0220	257730	>>>	NSD		Yes	
**sRNA2**	2313304	2313591	∼289	STM2213/STM2214	NT^m^	>>>	NSD		No	
**sRNA3**	2808084	2808210	∼127	STM2665/STM2667	2808135	>>>	STM2284	*glpA*: sn-glycerol-3-phosphate dehydrogenase	Yes	[R14]
**sRNA4**	3018904	3019048	∼145	STM2875/STM2876	NT	>>>	NSD		No	
**sRNA5**	4597115	4597181	∼71	STM4351/STM4355.S	NT	<><	STM1875	*yobA*: putative copper resistance protein	No	[R13]
**sRNA6**	3757015	3756884	∼132	STM3587/STM3588	3757010	<<<	NSD		Yes	
**sRNA7**	3275292	3275116	∼177	STM3114/STM3115	Not mapped	><<	STM0687	*ybfM*: putative outer membrane protein	No	[R19], [R20]
**sRNA8**	3240558	3240489	∼70	STM3078/STM3079.S	3240515	<<<	NSD		Yes	
**sRNA9**	757026	756967	∼60	STM0693/STM0694	NT	<<<	NSD		No	
**sRNA10**	679927	679828	∼100	STM0616/STM0617	679922	<<<	NSD		Yes	
**sRNA11**	139455	139727	273	STM0118/STM0119	NT	>>>	STM3954	*yigG*: putative inner membrane protein	No	[R15]
**sRNA12**	3733803	3733723	∼81	STM3564/STM3565	3733765	<<>	NSD		Yes	
**sRNA13**	1359947	1360181	∼235	STM1283/STM1284	NT	<><	NSD		No	
**sRNA14**	1415459	1415501	∼43	STM1337/STM1338	NT	>>>	NSD		No	
**sRNA15**	1691673	1691952	∼280	STM1601/STM1602	NT	<>>	NSD		No	
**sRNA16**	1334570	1334697	∼128	STM1249/STM1250	NT	<<>	STM0225	*hlpA*: periplasmic chaperone	No	
sRNA17	2905005	2905353	∼348	STM2762/STM2763	NT	<>>	STM0938	*ybjE*: putative inner membrane protein	*isrM* (Northern)	[28]
sRNA18	691922	691979	∼57	STM0627/STM0628	NT	<>>	STM1403	*sscB*: secretion system chaperone^$^	NT	[41]
sRNA19	2633992	2634070	∼78	STM2513/STM 2514	NT	<><	STM1426	*ribE*: riboflavin synthase alpha chain	NT	[R11]
sRNA20	4072486	4072617	∼131	STM3862/STM3863	NT	<><	STM2154	*mrp*: putative ATP-binding protein	*STnc410* (Predicted)	[23], [R12]
sRNA21	4561999	4562304	∼305	STM4316/STM4317	NT	<>>	STM4316	*STM4316*: putative cytoplasmic protein	NT	
sRNA22	3528698	3528642	∼56	STM3360/STM3361	NT	><>	STM3773	*STM3773*: putative transcriptional regulator	NT	
sRNA23	3474485	3474389	∼96	STM3305/STM3306	NT	><>	STM0244	*rcsF*: colanic acid synthesis regulator	NT	[43], [R14]
sRNA24	2116695	2116622	∼74	STM2037/STM2038	NT	<<>	STM4370	*yifI*: putative cytoplasmic protein	NT	
sRNA25	1627809	1627537	∼272	STM1551/STM1552	NT	<<>	STM3766	*STM3766*: putative cytoplasmic protein	NT	
sRNA26	75471	75555	∼84	STM0064/STM0066	NT	>>>	STM1379	*orf48*: putative amino acid permease	NT	
sRNA27	2077177	2077243	∼66	STM1994/STM1995	NT	<>>	STM4206	*STM4206*: putative phage glucose translocase	*rseX* (Northern)	[38], [39], [R16]
sRNA28	230161	230370	∼209	STM0194/STM0195	NT	>>>	STM0176	*stiB*: putative fimbrial chaperone	NT	[44]
sRNA29	4315449	4315163	∼286	STM4102/STM4103	NT	<<<	STM0335	*STM0335*: putative outer membrane protein	NT	
sRNA30	3598250	3597931	∼319	STM3445/STM3444	NT	<<<	STM3138	*mcpA*: putative methyl-accepting chemotaxis protein	NT	[R17]
sRNA31	3555129	3554959	∼170	STM3384/STM3383	NT	><>	STM4162	*thiF*: thiamine-biosynthetic protein	NT	[R18]
sRNA32	611107	610950	∼157	STM0550/STM0549	NT	<<<	STM3630	*dppA*: dipeptide transport protein	NT	[R21], [R22]
sRNA33	3528835	3528644	∼191	STM3361/STM3360	NT	><>	STM1417	*ssaP*: type III secretion system apparatus protein^$^	NT	[42]

a The sixteen sRNA candidates (sRNA1 – sRNA16 [shown in bold]) predicted by sRNAscanner **AND** identified in deep sequencing RNA libraries [37], [38] were chosen for experimental validation by Northern and 5′RACE analyses; five of these sixteen deep sequencing-supported hits, shown underlined, were also identified by TargetRNA. The remaining 17 sRNA candidates listed were associated with TargetRNA-identified putative mRNA targets.

b,c sRNAscanner-predicted transcript coordinates and length (nt).

d Genes flanking candidate sRNA loci obtained from KEGG genome maps.

e 5′ends of the primary transcripts identified using 5′RACE experiments.

f Stable transcripts identified by Northern analysis in this or other recent studies.

g The middle arrowhead represents the orientation of the sRNA gene; left and right arrowheads indicate orientations of flanking genes.

h Potential primary mRNA target identified using the TargetRNA tool [41].

i GenBank functional annotations of the putative target mRNAs.

l References relevant to the predicted target genes and/or the recently independently identified/predicted sRNAs; Full details of references indicated with ‘R’ are provided in Supporting Information (File S2).

m NT, denotes not tested.

### Northern and 5′ RACE based verification of novel sRNAs predicted by both sRNAscanner and deep sequencing

**Figure 6:**
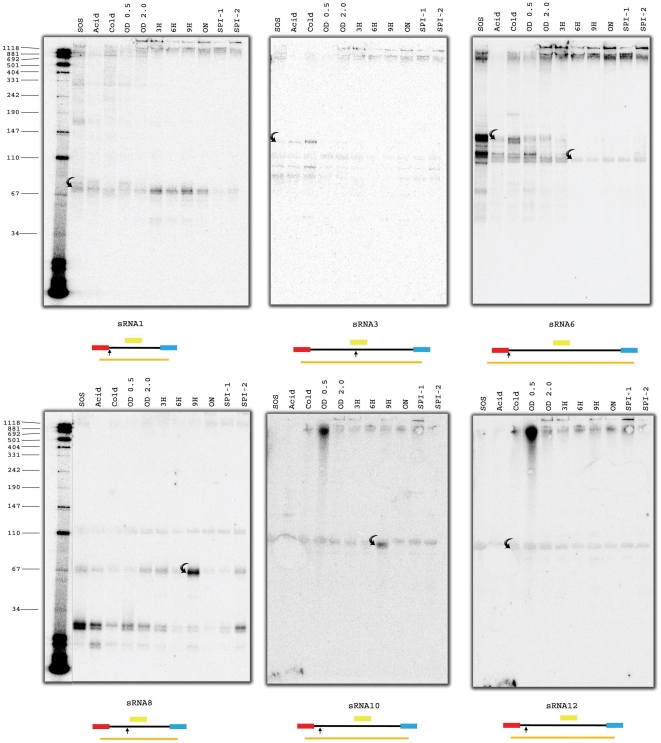
Total RNA was isolated from *Salmonella* Typhimurium SL1344 grown under eleven different conditions and subjected to Northern blotting using candidate sRNA-specific oligonucleotide probes. Details of growth conditions examined are outlined in the Materials and Methods section. The curved arrows indicate the six putative Northern-detected transcripts mapping to loci predicted by sRNAscanner. Additional bands seen for sRNA3, sRNA6 and sRNA8, are believed to represent degradation and/or processed forms of cognate sRNAs or overlapping mRNA transcripts. The to-scale schematics shown below each gel image indicate sRNAscanner-predicted TUs (red/black/blue), deep sequencing identified transcripts (orange line) and 5′RACE-defined transcript start-sites (vertical black arrow). The yellow boxes indicate the probes used to detect transcripts by Northern blot experiments. Red boxes represent putative promoter sequences; blue boxes indicated putative terminator sequences.

Northern blot experiments using oligonucleotide probes targeting the 16 novel sRNA candidates mentioned above were performed (Table S3). RNA samples were harvested from cells grown and/or subjected to eleven different growth conditions. Six of the candidates (sRNA1, sRNA3, sRNA6, sRNA8, sRNA10 and sRNA12) yielded distinct Northern-detectable transcripts of broadly similar sizes to the sRNAscanner-predicted entities (Figure 6). The additional non-specific bands seen with sRNA3-, sRNA6- and sRNA8-specific probes may comprise degraded and/or processed forms of the matching sRNAs or overlapping mRNA transcripts. Given the above assumption, sRNA1 and sRNA12 were expressed under all growth conditions tested; sRNA8 and sRNA10 were detected in late stationary phase samples only, whilst sRNA3 appeared to be induced specifically under cold shock conditions. The sRNAscanner-predicted sRNA6 overlapped with a previously proposed processed 5′UTR fragment of the *yhiI* transcript [38] that was likely to match the transcript we detected under ESP-2.0 conditions. However, in this study the sRNA6 locus was also found to express a distinct ∼70 nt transcript found under LSP and SPI-1/SPI-2 inducing conditions only.

The 5′ends of six candidate sRNA transcripts corresponding to the same Northern-supported candidates were successfully mapped by 5′RACE analysis. The 5′ RNA termini identified for sRNA1, sRNA6 and sRNA10 were coherent with computationally predicted transcriptional start sites but start-sites of the remaining three candidates varied significantly from those predicted by sRNAscanner (Table 2). The extents of overlap between sRNA predicted entities, deep sequencing identified sequences and 5′RACE mapped start-sites are shown schematically in Figure 6; Northern-detected transcripts were excluded as their precise locations could not be conclusively inferred on the basis of available data.

### Potential biological significance of sRNAscanner predictions for *Salmonella* Typhimurium

Recent discoveries of three sRNAscanner identified hits that had originally been classified as novel provide further biological validation of this algorithm; sRNA17, sRNA20 and sRNA29 are now known as *isrM* [29], *STnc410* [22] and *rseX* [39], [40], respectively. As many functionally characterized sRNAs are antisense regulators of cognate mRNA targets [41], we hypothesized that the presence of a matching TargetRNA hit may allow for more reliable identification of genuine sRNAs. However, we emphasize that bioinformatically-derived predictions of sRNA–mRNA interactions remain fraught with problems. Consequently, pending experimental validation by gel-shift assays or other methodologies TargetRNA data need to be treated as truly putative. We identified 22 sRNAscanner hits with TargetRNA-identified potential mRNA targets (Figure S5); five had also been detected in the deep sequencing dataset (Table 2). Several TargetRNA-identified genes play roles in pathogenesis. sRNA18 putatively targets STM1403 that codes for SscB, a type III secretion system (T3SS) chaperone encoded by *Salmonella* pathogenicity island 2 (SPI-2). SscB is needed for normal secretion and function of the SseF T3SS effector, which in turn is required for *Salmonella*-induced epithelial cell filamentation and bacterial proliferation in macrophages [42]. sRNA33 is believed to regulate *ssaP*, which is postulated to code for part of the SPI-2 T3SS translocon apparatus itself [43]. sRNA23 is predicted to regulate RcsF which has been proposed as one of two proximal membrane-located sensors for the Rcs phosphorelay signal transduction system that coordinately regulates expression of SPI-1/SPI-2, flagellar, fimbrial and capsule-related colonic acid synthesis genes [44]. sRNA28 is hypothesized to target *stiB*, a fimbrial chaperone gene, potentially allowing for sRNA28-based fine-tuning of Sti fimbriae expression [45]. sRNAs have also been shown to regulate S. *Typhimurium* outer membrane protein (OMP) profiles in response to envelope stress [46] or nutrient availability [39]. Similarly, sRNA29 and sRNA7 are predicted to interact with OMP-encoding genes (Table 2). Clearly, data supported solely by sRNAscanner and TargetRNA bioinformatics predictions remain speculative and robust experimentation would be required to validate these prior to drawing firm conclusions.

### Conclusions

We have developed and implemented a simple PWM-based strategy for the discovery of intergenic sRNA genes. Despite use of a small, single species-derived training set, we have demonstrated the major utility of sRNAscanner to predict large numbers of potential sRNA genes in diverse bacterial species. Undoubtedly, it is vital to further experimentally validate the predictive accuracy of sRNAscanner and other sRNA prediction programmes using Northern blot analysis, ultra-high-density cDNA sequencing [37], [38] and other emerging tools. Nevertheless, caution is advisable in interpretation of results as each experimental method has its own strengths and weaknesses. Furthermore, transcriptional signals would be expected to vary considerably between phylogenetically distant organisms. Consistent with this idea, we found that the *E. coli*-derived PWMs used in this study performed well with medium and low GC genomes but not with high GC genomes. Consequently, we propose that an organism-targeted approach is likely to lead to significantly enhanced performance characteristics. Importantly the tool developed and the strategy proposed would allow users to generate individualized PWMs based on species-, genus- or family-derived training sets to better identify sRNA genes in selected bacterial organisms. In addition, a reiterative process of PWM optimization and selection of rationally informed cut-offs based on newly discovered and validated sRNAs may allow for progressively higher levels of specificity without excessive loss of sensitivity. Finally, we propose that PWM-based scanning strategies may in time prove to be a powerful way of revealing other cryptic codes not only in DNA but in protein molecules as well.

## Supporting Information

Table S1 Details of sRNAscanner training dataset.
(0.03 MB PDF)

Table S2 List of known E. coli K-12 MG1655 sRNA TUs identified by sRNAscanner.
(0.08 MB PDF)

Table S3 Oligonucleotides used in this study.
(0.02 MB PDF)

Table S4 Details of known and novel sRNA regions predicted by sRNAscanner in 13 bacterial genomes.
(0.02 MB PDF)

Table S5 Analysis of Virtual Intergenic Genome Sequences (VIGS) and Random Intergenic Genome Sequences (RIGS) derived from the E. coli K-12 genome using sRNAscanner and Glimmer.
(0.03 MB PDF)

Figure S1 Training set-derived PWM1 - PWM3 matrices.
(0.04 MB PDF)

Figure S2 R1 versions of random matrices.
(0.03 MB PDF)

Figure S3 R2 versions of random matrices
(0.03 MB PDF)

Figure S4 R3 versions of random matrices.
(0.03 MB PDF)

Figure S5 TargetRNA-identified putative sRNA-mRNA interactions.
(0.07 MB PDF)

File S1 Details of known and novel sRNAs predicted by sRNAscanner in the 13 genomes analysed.
(0.47 MB XLS)

File S2 Supplementary References.
(0.03 MB PDF)
