# Correction: sRNAscanner: A Computational Tool for Intergenic Small RNA Detection in Bacterial Genomes

**DOI:** 10.1371/annotation/71408e55-e1d3-4950-9c3b-d3a3ad66a1ff

**Published:** 2010-09-16

**Authors:** Jayavel Sridhar, Narmada Sambaturu, Radhakrishna Sabarinathan, Hong-Yu Ou, Zixin Deng, Kanagaraj Sekar, Ziauddin Ahamed Rafi, Kumar Rajakumar

The second author's name was entered incorrectly. The correct name is Narmada Sambaturu. The correct citation is: Sridhar J, Sambaturu N, Sabarinathan R, Ou H-Y, Deng Z, et al. (2010) sRNAscanner: A Computational Tool for Intergenic Small RNA Detection in Bacterial Genomes. PLoS ONE 5(8): e11970. doi:10.1371/journal.pone.0011970

