# Supplementary material for: sRNAscanner: A Computational Tool for Intergenic Small RNA Detection in Bacterial Genomes
Source: PLoS One. 2010 Aug 5;5(8):e11970. doi: 10.1371/journal.pone.0011970 (PMC2916834; doi:10.1371/journal.pone.0011970)
Supplement: Table S1 — Details of sRNAscanner training dataset. (0.03 MB PDF) [file pone.0011970.s001.pdf]

Table S1a. Base distribution in -35 and -10 regions of mRNA and sRNA promoters

mRNA promoters compiled by Harley and Reynolds 1987 [R23]												
	T	T	G	A	C	A	T	A	T	A	A	T
T	78	82	15	20	10	24	82	7	52	14	19	89
G	10	5	68	10	7	17	7	1	12	15	11	2
C	9	3	14	13	52	5	8	3	10	12	21	5
A	3	10	3	58	32	54	3	89	26	59	49	3
Small RNA specific promoters compiled from this study												
	T	T	G	A	C	A	T	A	T	A	A	T
T	70	70	20	30	20	20	90	10	60	30	10	100
G	10	0	60	0	10	10	10	0	10	10	20	0
C	10	10	0	0	60	30	0	0	20	0	20	0
A	10	20	20	70	10	40	0	90	10	60	50	0

Table S1b. Previously defined sRNA-specific transcriptional signals used as the training set to generate PWM1 and PWM2.

**-35 box[a]**	**-10 box[b]**	**sRNA[c]**	**strand[d]**	**Reference**
TTGTAA	TTCTAT	*csrB*	<<<	[R1]
TTGTCA	TAAGGT	*dsrA*	<<<	[R2]
CTGATC	TATATT	*gadY*	>>>	[R3]
TTGAGC	TATAGT	*gcvB*	>>>	[R4]
TATACT	TATTCT	*micC*	>>>	[R5]
AAAACA	TAGAAT	*micF*	>>>	[R6]
TTATCC	GATAAT	*oxyS*	<<<	[R7]
GTGACA	TATACT	*rnpB*	<<<	[R8]
TCGACG	TACTAT	*rprA*	>>>	[R9]
TTTATT	TATAAT	*rydC*	<<<	[R10]

[a] Experimentally identified sRNA-specific [-35] promoter box.
[b] Experimentally identified sRNA-specific [-10] promoter box.
[c] Well characterized *E. coli* K12-MG1655 sRNA genes selected for the training set.
[d] Orientation of sRNA gene with respect to flanking genes and genome.

Table S1c. Rho-independent terminator sequences collected from experimentally confirmed sRNAs in *E. coli* K12 [21]

>NC_000913.2_1550372_1550402_eco_c0362
TGTTGCGTTTGTCATCAGTTCTAAATGGCGC
>NC_000913.2_3119518_3119548_eco_c0719
GGCCCGCTAGTAATGCGCTACGGGTATTTAA
>NC_000913.2_2940898_2940928_eco_gcvB
ACCGCCTAATTGCGGTGCTTTTTTTTACCTT
>NC_000913.2_2651715_2651745_eco_IS128
AAATCAGGCGGTTTTTTGTTGCTGGTCCGGT
>NC_000913.2_1435229_1435259_eco_micC
CCGAACAGTCGTCCGGGCTTTTTTTTTAGAA
>NC_000913.2_2311171_2311201_eco_micF
ACCGGATGCCTCGCATTCGGTTTTTTTTACC
>NC_000913.2_1768476_1768506_eco_rprA
CCATCTCCCACGATGGGCTTTTTTTTAACAT
>NC_000913.2_1921335_1921365_eco_ryeA
GTGAAAGAACTGAGGCGGTTTTTTTATTGGA
>NC_000913.2_2151348_2151378_eco_ryeC
AGGTTTCCCCCTCCCCCTGGTGTTCTTAGTA
>NC_000913.2_3054988_3055018_eco_rygC
GCCCTCGCTTCGGTGAGGGCTTTACCGTTAC
>NC_000913.2_2689598_2689628_eco_tke1
AAGCTCCCGGAGTTGGGAGCTTATGATAGTG
>2069510-2069540_eco_isrC_IS102
CCACGTTTGTGGGTACCGGCTTTTTTATTCA
>3236105-3236135_eco_sraF
TGTGCTTCGTGCCGCGTGGCAGCCAAATGCA
>3662574-3662604_eco_gadY
ATTTTTTGCTGTTCATGATAAATATCGAATG
>NC_000913.2_2023239_2023269_eco_dsrA
CCCCTCAGGGTCGGGATTTTTTTATTGTGCA
>NC_000913.2_1762690_1762720_eco_rydB
GTCGCACAGACTTAAGGGTTTTCTTATTTCT
>NC_000913.2_1489461_1489491_eco_rydC
TCGGTTTTAGTACAGGCGTTTTCTTTAAATA
>NC_000913.2_2651687_2651717_eco_ryfA
TTTTGGCGGTTTTTTGTTTTTTAAAGGAAAG
>gi|49175990:c1403706-1403676_eco_isrA
GGATGTCGACAGACTCTATTTTTTTATGCAG
>gi|49175990:c3192759-3192729_eco_ryD
TTCGGGAGGGGCTTTCCCGTTTCAGCCCCCT
>c3578579-3578549_sraI
CGCGCGCTGGAAGCCGGGAAAAATGTGCTGG
