# Supplementary material for: sRNAscanner: A Computational Tool for Intergenic Small RNA Detection in Bacterial Genomes
Source: PLoS One. 2010 Aug 5;5(8):e11970. doi: 10.1371/journal.pone.0011970 (PMC2916834; doi:10.1371/journal.pone.0011970)
Supplement: Table S2 — List of known E. coli K-12 MG1655 sRNA TUs identified by sRNAscanner. (0.08 MB PDF) [file pone.0011970.s002.pdf]

Table S2. List of sixty six known *E. coli* K12-MG1655 sRNA TUs that were identified by sRNAscanner.

Start1[a]	*D1*[b]	*D2*[c]	*End3*[d]	*Score1*[e]	*Score2*[f]	*Score3*[g]	*CSS*[h]	*Strand*[i]	*Comment*[j]
111	12	151	316	4.34949	2.91965	8.24484	15.514	p	*Thr_leader*
17035	18	49	17144	2.05755	5.37638	5.88234	13.3163	p	*sokC*
77321	16	218	77597	2.05755	4.59522	7.4388	14.0916	p	*sgrS*
83929	13	308	83567	3.32784	3.57357	8.94716	15.84857	c	*Leu_leader*
123005	16	50	122898	2.52555	2.52761	7.46332	12.51648	c	*tp2*
686023	13	69	685900	3.16148	2.35325	5.63453	11.14926	c	*sroC*
887288	16	44	887187	2.30619	2.41939	4.3023	9.02788	c	*rybB*
1143676	18	325	1144061	1.78929	1.96413	6.06757	9.82099	p	*rne5*
1145823	17	103	1145985	2.89322	2.41939	4.42801	9.74062	p	*sraB_PsrD*
1229971	14	44	1229872	2.15777	2.91965	4.70722	9.78464	c	*C0299*
1268516	12	48	1268618	3.22762	4.52908	7.36021	15.1169	p	*rdlA*
1269051	12	48	1269153	3.22762	4.52908	7.36021	15.1169	p	*rdlB*
1269586	12	48	1269688	3.22762	4.52908	7.36021	15.1169	p	*rdlC*
1286580	15	233	1286870	3.37605	3.37499	4.51164	11.2627	p	*rtt*
1321167	17	123	1320986	4.98548	1.57218	10.3006	16.85826	c	*Trp_leader*
1403698	12	240	1403992	3.22762	4.16136	4.46953	11.8585	p	*IS061_isrA*
1403838	17	105	1403675	1.93771	2.41939	6.46964	10.82674	c	*IS061*
1416309	16	240	1416607	2.59164	4.16136	7.85388	14.6069	p	*dicF*
1435036	18	163	1435259	2.67391	4.02883	8.77217	15.4749	p	*micC*
1489561	15	45	1489460	2.30619	6.03031	7.95298	16.28948	c	*rydC*
1647347	14	68	1647471	4.18314	1.57215	5.70936	11.4647	p	*dicF*
1762888	17	141	1762689	2.94218	2.59375	6.94529	12.48122	c	*rydB*
1768361	17	86	1768506	2.00385	4.35473	9.29241	15.651	p	*rprA*
1903414	17	217	1903690	3.16148	4.59522	5.77931	13.536	p	*yybP_YkoY*
1921041	18	264	1921365	2.35439	3.57357	6.39807	12.326	p	*sraC_ryeA*
1956288	13	40	1956194	2.20597	4.52908	5.25889	11.99394	c	*ryeF*
1986058	16	140	1985861	3.32784	5.37638	5.29614	14.00036	c	*IS092_isrB*
2023396	14	101	2023240	2.67391	3.94136	10.6813	17.29657	c	*dsrA*
2031614	17	95	2031768	3.96383	4.02883	7.47558	15.4682	p	*rseX*
2069307	17	174	2069540	3.91487	4.42087	8.43539	16.7711	p	*IS102_isrC*
2087914	12	204	2088172	2.79376	4.42087	5.82731	13.0419	p	*His_Leader*
2151167	15	158	2151382	2.20597	2.81143	5.82675	10.8441	p	*QUAD1a*
2151634	17	120	2151813	4.98548	2.91965	4.3799	12.285	p	*QUAD1b*
2165060	14	112	2165228	1.56998	2.41939	5.19922	9.18859	p	*ryeE*
2311116	16	179	2311353	3.32784	1.57215	6.17275	11.0727	p	*micF*
2651506	16	174	2651738	4.18314	4.52908	6.219	14.9312	p	*IS128*
2651640	16	40	2651738	2.35439	2.59375	6.219	11.1671	p	*ryfA*
2689131	14	133	2689320	2.42528	1.57213	4.72639	8.7238	p	*tke1*
2689441	13	216	2689171	2.52549	6.03031	5.13033	13.68613	c	*tke1_glmY*
2698120	14	314	2698490	1.71845	2.31673	5.02186	9.05704	p	*ryfB*
2698505	18	57	2698622	2.57375	2.41939	8.78396	13.7771	p	*ryfC*

2744415	17	136	2744221	2.42528	2.55192	7.47016	12.44736	c	*rpsP 5'*
2753699	14	306	2754061	1.78929	2.55192	8.43406	12.7753	p	*tmRNA*
2885267	15	268	2885592	1.93771	3.94138	4.91637	10.7955	p	*sokX*
2922580	17	348	2922174	2.57379	2.52761	5.43862	10.54002	c	*csrB*
2940683	17	186	2940928	3.10778	5.18301	9.27923	17.57	p	*gcvB*
2967539	13	197	2967288	2.89322	2.41939	4.62762	9.94023	c	*sraE_omrA_omrB*
2974292	18	114	2974119	3.96383	1.57212	8.70576	14.24171	c	*sraE_omrA_omrB*
3054837	17	201	3055097	4.98548	2.33186	4.83957	12.1569	p	*QUAD1c/rygC*
3119318	12	281	3119653	2.52553	5.18301	4.51171	12.2203	p	*C0719*
3182845	15	290	3182499	2.20597	1.57209	4.87838	8.65644	c	*sroG*
3192921	17	129	3192734	3.76171	3.57357	4.10227	11.43755	c	*QUAD1d_rygD*
3193204	16	250	3192897	3.96383	4.42087	5.40649	13.79119	c	*QUAD1e_rygE*
3236360	17	171	3236590	4.34949	2.91965	8.45355	15.7227	p	*yybP_ykoY_psrN*
3268402	13	151	3268197	2.20597	3.57357	5.00101	10.78055	c	*RNaseP_bact_a*
3309786	12	305	3309428	3.96383	4.16136	4.71052	12.83571	c	*S15*
3579074	18	76	3578939	1.61818	5.37638	10.1226	17.11716	c	*ryhB_SraI*
3662852	17	86	3662997	1.93771	4.59522	9.1865	15.7194	p	*gadY*
3698129	12	48	3698231	3.22762	4.52908	7.22135	14.9781	p	*rdlD*
3706755	17	101	3706596	3.76171	2.81143	6.20238	12.77552	c	*rttR*
3851074	17	169	3850847	3.54715	1.76547	6.46886	11.78148	c	*lstR-2*
3851226	16	322	3850847	2.15777	4.16136	6.46886	12.78799	c	*lstR-1*
3984402	12	210	3984666	2.20597	1.76546	7.35598	11.3274	p	*sraJ_glmZ*
4047915	15	63	4048035	1.78929	2.81143	5.71214	10.3129	p	*Spot_42*
4049024	16	225	4049307	2.52551	3.57357	5.66983	11.7689	p	*csrC*
4156454	18	100	4156295	2.89322	4.02883	6.48072	13.40277	c	*oxyS*

[a] Start of the sRNAscanner-predicted transcriptional unit (TU).
[b] Length of the sequence-independent spacer between the identified [-35] and [-10] promoter boxes.
[c] Predicted length of sRNA transcript-encoding region.
[d] End coordinate of the predicted sRNA TU.
[e] Sum of scores (SS) value for the identified orphan [-35] promoter box.
[f] Sum of scores (SS) value for the identified orphan [-10] promoter box.
[g] Sum of scores (SS) value for the identified orphan rho-independent terminator.
[h] Cumulative sum of scores (CSS) value of the predicted sRNA TU.
[I] The coding strand of the predicted sRNA TU; p, positive; c, complementary.
[j] Identity of the sRNAscanner-predicted known *E. coli* K12-MG1655 sRNA TU as recorded in the Rfam [34] and/or sRNAMap [1] databases based on their location coordinates and orientation.
