# Supplementary material for: sRNAscanner: A Computational Tool for Intergenic Small RNA Detection in Bacterial Genomes
Source: PLoS One. 2010 Aug 5;5(8):e11970. doi: 10.1371/journal.pone.0011970 (PMC2916834; doi:10.1371/journal.pone.0011970)
Supplement: Table S3 — Oligonucleotides used in this study. (0.02 MB PDF) [file pone.0011970.s003.pdf]

Table S3. List of deoxyoligonucleotides used in this study

sRNA	***Oligo***	***Sequence***
sRNA 1	JVO5718	5’-GCGCTTTGTACACCACTCAT-3’
sRNA 2	JVO5719	5’-GATTACCCGATTGTTGCTGT-3’
sRNA 3	JVO5720	5’-TTCTTTATCACATGACGAAACG-3’
sRNA 4	JVO5721	5’-ACGAATTCTTACGCTGTGAATA-3’
sRNA 5	JVO5722	5’-AGGAATAGTCAATAAAGCTAACGT-3’
sRNA 6	JVO5723	5’-TAATGTTGTCATCATCAGCGTT-3’
sRNA 7	JVO5724	5’-AGAAATTTCGCGCAATCAT-3’
sRNA 8	JVO5725	5’-ACGAGGAAGGGTTTGAATTT-3’
sRNA 9	JVO5726	5’-TGGTTTTGCCTGCAAATTCT-3’
sRNA 10	JVO5727	5’-TTGAAGTGATTTAGTTCACATTCG-3’
sRNA 11	JVO5728	5’-ACATATTGCACCGTGTTAACAG-3’
sRNA 12	JVO5729	5’-CGGAATAATTCACTAGCATTCAG-3’
sRNA 13	JVO5730	5’-CATAGCAGACGACGTGAACC-3’
sRNA 14	JVO5731	5’-ATGCTTGCGTTACCTTATGTG-3’
sRNA 15	JVO5732	5’-GTAACGATTAGCTGACGGCTT-3’
sRNA 16	JVO5733	5’-AAACGAGACAGTGAACAGTACC-3’
A4 adapter oligo	JVO-0367	5’-ACTGACATGGAGGAGGGA-3’
pCR2.1 TOPO	M13-forward	5´-GTAAAACGACGGCCAG-3´
pCR2.1 TOPO	M13-reverse	5´-CAGGAAACAGCTATGAC-3´
