# Supplementary material for: sRNAscanner: A Computational Tool for Intergenic Small RNA Detection in Bacterial Genomes
Source: PLoS One. 2010 Aug 5;5(8):e11970. doi: 10.1371/journal.pone.0011970 (PMC2916834; doi:10.1371/journal.pone.0011970)
Supplement: Table S4 — Details of known and novel sRNA regions predicted by sRNAscanner in 13 bacterial genomes. (0.02 MB PDF) [file pone.0011970.s004.pdf]

Table S4. A complete list of known and novel sRNA regions predicted by sRNAscanner.

**Bacterial strain (GenBank Acc. No)**	*Known* (CSS > 14)	*Novel* (CSS > 14)	*Reference*
Bacillus anthracis Ames (AE016879)	49	535	File S1 Sheet 1-2
Clostridium tetani E88 (AE015927)	27	285	File S1 Sheet 3-4
Chlamydia trachomatis D/UW-3/Cxa (AE001273)	1	27	File S1 Sheet 5-6
Helicobacter pylori 26695 (AE000511)	2	107	File S1 Sheet 7-8
Mycobacterium tuberculosis CDC1551 (AE000516)	0	1	File S1 Sheet 9-10
Pseudomonas aeruginosa PAO1 (AE004091)	3	17	File S1 Sheet 11-12
Salmonella enterica Typhi CT18 (AL513382)	15	175	File S1 Sheet 13-14
Staphylococcus aureus N315 (BA000018)	17	253	File S1 Sheet 15-16
Streptococcus pneumoniae TIGR4 (AE005672)	9	190	File S1 Sheet 17-18
Streptococcus pyogenes M1 GAS (AE004092)	4	162	File S1 Sheet 19-20
Yersinia pestis KIMa (AE009952)	7	287	File S1 Sheet 21-22
Salmonella enterica Typhimurium LT2 (NC_003197)	24	135	File S1 Sheet 23-24
Escherichia coli K12-MG1655 (NC_000913)	22	170	File S1 Sheet 25-26
