# Supplementary material for: sRNAscanner: A Computational Tool for Intergenic Small RNA Detection in Bacterial Genomes
Source: PLoS One. 2010 Aug 5;5(8):e11970. doi: 10.1371/journal.pone.0011970 (PMC2916834; doi:10.1371/journal.pone.0011970)
Supplement: Table S5 — Analysis of Virtual Intergenic Genome Sequences (VIGS) and Random Intergenic Genome Sequences (RIGS) derived from the E. coli K-12 genome using sRNAscanner and Glimmer. (0.03 MB PDF) [file pone.0011970.s005.pdf]

Table S5. A novel approach to qualitatively assess the specificity of sRNAscanner using Virtual Intergenic Genome Sequences (VIGS) and Random Intergenic Genome Sequences (RIGS) derived from the *E. coli* K-12 genome.

**Sequence** [a]	**sRNAscanner**		**Glimmer-3.02**[d]		
	**No. of TU predicted** [b]	**$TU_{VIGS/RIGS}$** [c]	**Sequence** [e]	**No. of CDS predicted** [f]	**CDS > 1 kb** [g]
E. coli K12	17 Known, 119 Novel (136)	-			
E. coli K12_VIGS	17 Known, 554 Novel (571)	435	*E. coli* K12	4395	1201
K12_RIGS-1	17 Known, 279 Novel (296)	160	R1[$]	6804	2
K12_RIGS-2	17 Known, 291 Novel (308)	172	R2[$]	6494	3
K12_RIGS-3	17 Known, 266 Novel (283)	147	R3[$]	5853	1
K12_RIGS-4	17 Known, 274 Novel (291)	155	R4[$]	6439	4
K12_RIGS-5	17 Known, 264 Novel (281)	145	R5[$]	6836	2
K12_RIGS-6	17 Known, 255 Novel (272)	136	R6[$]	6887	3
K12_RIGS-7	17 Known, 270 Novel (287)	151	R7[$]	5853	2
K12_RIGS-8	17 Known, 270 Novel (287)	151	R8[$]	6153	4
K12_RIGS-9	17 Known, 277 Novel (294)	158	R9[$]	5830	4
K12_RIGS-10	17 Known, 265 Novel (282)	146	R10[$]	6486	6
	Mean: 288, Standard Deviation (S.D): 9.87 [RIGS only]	Mean: 152, S.D: 9.87 [RIGS only]		Mean: 6363, S.D: 86.6 (R1-R10 only)	Mean: 3.01, S.D:2.05 (R1-R10 only)

[a] *E. coli* K12 (4639675 bp) sequence with VIGS, RIGS-1, RIGS-2, ….., RIGS-10 added to the end of the native *E. coli* K12 sequence. The ptt file used ensured that the appended sequences (VIGS, RIGS-1, RIGS-2, ….) were treated as non-coding and that no CDS mapped to these sequences. 'True' intergenic sequences was extracted from the complete genome of K12 (NC_000913.fna) by excluding CDS (from *.ptt file) and t/r/s/RNA (from *.rnt) annotations and concatenated end-to-end in their native order to generate the VIGS. The total length of the VIGS was 640895 bp. To generate the random variants, the VIGS was randomly shuffled using the emboss-shuffleseq application for 10 independent iterations using automated scripts, to produce RIGS1, RIGS2………..RIGS-10. The created VIGS and RIGS-x genome fractions were appended to the end of the *E. coli* K12 genome to generate hybrids that were 5280570 bp in size.
[b] Total number of intergenic TU indentified within the entire hybrid sequences when analysed by the most recent version of sRNAscanner (CSS >14).
[c] Total number of intergenic TU indentified within the VIGS or RIGS regions alone. We hypothesized that the ratio of these numbers would provide a qualitative measure of the specificity of sRNAscanner.
[d] CDS/ORFs were predicted using the Glimmer-3.02 (http://www.cbcb.umd.edu/software/glimmer/) [R24] tool.
[e] K12 genome (4639675 bp) was randomly shuffled, whilst maintaining an identical nucleotide frequency distribution and length. Ten different Random Genomes (R1..R10) were generated.
[f] The total number of CDS predicted within the K12 and random genomes are shown. [g] The number of predicted CDS > 1 kb are shown. These data were derived to emphasize the impact of randomizing genome sequences.
