# Supplementary material for: sRNAscanner: A Computational Tool for Intergenic Small RNA Detection in Bacterial Genomes
Source: PLoS One. 2010 Aug 5;5(8):e11970. doi: 10.1371/journal.pone.0011970 (PMC2916834; doi:10.1371/journal.pone.0011970)
Supplement: Figure S1 — Training set-derived PWM1 - PWM3 matrices. (0.04 MB PDF) [file pone.0011970.s006.pdf]

(A) PWM1-training

```
A| -0.788457| -0.200671| -0.200671|  0.969401| -0.788457|  0.435318|
C| -0.788457| -0.788457|   -2.3979|   -2.3979|  0.820981|  0.167054|
G| -0.788457|   -2.3979|  0.820981|   -2.3979| -0.788457| -0.788457|
T|  0.969401|  0.969401| -0.200671|  0.167054| -0.200671| -0.200671|
```

(B) PWM2-training

```
A|   -2.3979|   1.21302| -0.788457|  0.820981|  0.646627|   -2.3979|
C|   -2.3979|   -2.3979| -0.200671|   -2.3979| -0.200671|   -2.3979|
G| -0.788457|   -2.3979| -0.788457| -0.788457| -0.200671|   -2.3979|
T|   1.21302| -0.788457|  0.820981|  0.167054| -0.788457|   1.31568|
```

(C) PWM3-training

```
A|0.127833|-0.893818|-0.257829|-0.893818|-0.893818|-0.257829|-0.526093|
 -0.526093| -0.04652|-0.893818|-0.526093|-0.526093|-0.257829|-0.526093|
 -0.257829|-0.893818|-  1.4816|-0.526093|  -1.4816|-0.526093|-0.526093|
 -0.893818|-0.257829| 0.127833|-0.526093| 0.127833| -0.04652| 0.276253|
 -0.257829| 0.127833| 0.405465|

C| -0.04652| 0.405465| 0.405465| -0.04652| 0.276253| 0.127833| 0.405465|
   -0.04652| -0.04652| -0.04652|-0.257829|-0.257829| 0.127833| 0.127833|
  -0.526093| 0.276253|-0.257829| 0.127833| -3.09104|-0.257829|-0.893818|
    -1.4816|-0.257829|-  1.4816|-0.893818|-0.526093|-0.893818|-0.257829|
   -0.04652| 0.127833|-0.526093|
G|- 0.04652| 0.127833| -0.04652| 0.276253|0.127833|-0.257829|-0.257829|
   0.405465| 0.405465| 0.276253| 0.276253|0.276253| -0.04652|-0.257829|
   0.519875| 0.405465|  0.62253| 0.519875|0.276253|-0.893818|  -1.4816|
  -0.526093|  -1.4816|-0.893818|-0.257829| -1.4816| 0.127833|-0.893818|
   0.276253|-0.257829| -0.04652|
T|-0.04652| -0.04652|-0.257829| 0.276253|0.127833|0.276253|0.127833|
  -0.04652|-0.526093| 0.276253| 0.276253|0.276253|0.127833|0.405465|
  -0.04652|-0.257829| 0.127833|-0.526093|0.879249|0.800778| 1.01983|
   1.01983| 0.800778| 0.800778| 0.800778| 0.71562|0.405465|0.405465|
  -0.04652| -0.04652| -0.04652|
```

Figure S1. Position weight matrices constructed using previously defined transcription signal data for ten known *E. coli* K12 MG1655 sRNA genes (see Table S1 for details). **(A)** PWM1 constructed using –35 promoter box data. **(B)** PWM2 constructed using –10 promoter box data. **(C)** PWM3 constructed using Rho-independent terminator box data. The above three training set-derived PWM were used in the analyses described in this study.
