# Supplementary material for: sRNAscanner: A Computational Tool for Intergenic Small RNA Detection in Bacterial Genomes
Source: PLoS One. 2010 Aug 5;5(8):e11970. doi: 10.1371/journal.pone.0011970 (PMC2916834; doi:10.1371/journal.pone.0011970)
Supplement: Figure S2 — R1 versions of random matrices. (0.03 MB PDF) [file pone.0011970.s007.pdf]

(A) PWM1-R1

```
A|0.435318|0.969401|-0.200671|-0.200671|-0.788457|-0.788457|
C|0.167054| -2.3979|-0.788457|  -2.3979| 0.820981|-0.788457|
G|-0.78845| -2.3979|  -2.3979| 0.820981|-0.788457|-0.788457|
T|-0.20067|0.167054| 0.969401|-0.200671|-0.200671| 0.969401|
```

(B) PWM2-R1

```
A| 0.646627|  1.21302|  -2.3979|-2.3979|-0.788457| 0.820981|
C|-0.200671|  -2.3979|  -2.3979|-2.3979|-0.200671|  -2.3979|
G|-0.200671|  -2.3979|-0.788457|-2.3979|-0.788457|-0.788457|
T|-0.788457|-0.788457|  1.21302|1.31568| 0.820981| 0.167054|
```

(C) PWM3-R1

```
A|-0.893818|-0.526093|-0.526093| -0.04652|-0.893818|-0.257829|-0.257829|
  -0.526093| 0.127833| 0.127833| 0.405465|-0.526093|-0.526093|-0.257829|
  -0.893818|-0.893818| 0.127833|-0.526093|-0.257829|-0.257829| -0.04652|
    -1.4816|  -1.4816|-0.893818|-0.257829| 0.127833| 0.276253|-0.526093|
  -0.893818|-0.526093|-0.526093|
C|-1.4816| 0.127833|-0.257829|-0.893818| -0.04652| 0.127833| 0.127833|
 0.405465|  -1.4816|-0.526093|-0.526093| -0.04652|-0.893818| 0.405465|
 -0.04652| 0.276253| 0.127833|-0.257829|-0.526093| -0.04652| -0.04652|
 -3.09104|-0.257829| 0.276253|-0.257829| -0.04652|-0.257829|-0.893818|
 0.405465| 0.127833|-0.257829|
G|-0.526093|-0.257829|-0.893818|0.127833|0.276253| -0.04652|-0.257829|
  -0.257829|-0.893818|  -1.4816|-0.04652|0.405465|-0.257829| -0.04652|
   0.276253| 0.127833|-0.257829|0.276253|0.519875| 0.276253| 0.405465|
   0.276253|  0.62253| 0.405465| -1.4816|-0.04652|-0.893818|  -1.4816|
   0.127833| 0.519875| 0.276253|
T| 1.01983| 0.405465| 0.800778|0.405465|0.276253|0.127833| 0.276253|
  0.127833| 0.800778|  0.71562|-0.04652|-0.04652|0.800778|-0.257829|
  0.276253| 0.127833| -0.04652|0.276253|-0.04652|-0.04652|-0.526093|
  0.879249| 0.127833|-0.257829|0.800778|-0.04652|0.405465|  1.01983|
  -0.04652|-0.526093| 0.276253|
```

Figure S2. The R1 versions of random position weight matrices constructed by randomly shuffling columns within individual genuine matrices. **(A)** PWM1-R1 constructed from PWM1. **(B)** PWM2-R1 constructed from PWM2. **(C)** PWM3-R1 constructed from PWM3.
