# Supplementary material for: sRNAscanner: A Computational Tool for Intergenic Small RNA Detection in Bacterial Genomes
Source: PLoS One. 2010 Aug 5;5(8):e11970. doi: 10.1371/journal.pone.0011970 (PMC2916834; doi:10.1371/journal.pone.0011970)
Supplement: Figure S3 — R2 versions of random matrices (0.03 MB PDF) [file pone.0011970.s008.pdf]

(A) PWM1-R2

```
A|-0.788457|   -2.3979|-0.200671|  -2.3979|-0.788457|  0.167054|
C|-0.788457|-0.200671|-0.200671|0.969401|-0.200671|  0.435318|
G|0  .969401|  0.969401|  0.820981|0.167054|  0.820981|-0.788457|
T|-0.788457|-0.788457|   -2.3979|  -2.3979|-0.788457|-0.200671|
```

(B) PWM2-R2

```
A|   1.21302|   -2.3979|-0.788457|-0.788457|  0.200671|1.31568|
C|   -2.3979|   1.21302|-0.200671|  0.820981|-0.788457|-2.3979|
G|   -2.3979|   -2.3979|-0.788457|   -2.3979|-0.200671|-2.3979|
T|-0.788457|-0.788457|  0.820981|  0.167054|  0.646627|-2.3979|
```

(C) PWM3-R2

```
A |-0.04652|-0.893818|-0.257829| 0.276253|0.127833|-0.257829|-0.526093|
    -0.04652| 0.405465|-0.893818| 0.276253|0.276253| 0.127833| 0.405465|
   -0.257829|-0.257829| 0.127833| 0.519875| -1.4816|-0.893818|-0.526093|
     1.01983|-0.257829| 0.127833|-0.893818| -1.4816| -0.04652|-0.257829|
   -0.257829| -0.04652| -0.04652|
C |-0.04652| -0.04652| -0.04652| 0.276253| 0.276253|-0.257829|0.127833|
    -0.04652|-0.526093| -0.04652| 0.276253|-0.526093| -0.04652|0.127833|
   -0.526093| 0.276253|-0.257829| 0.127833| -3.09104|-0.257829| 1.01983|
   -0.526093| 0.800778|-0.893818|-0.526093|  0.71562| 0.127833|0.276253|
    -0.04652| 0.127833|-0.526093|
G |0.127833| 0.405465|-0.257829| -0.04652|0.127833| 0.276253| 0.405465|
    0.405465| -0.04652| 0.276253|-0.257829|0.276253| 0.127833|-0.257829|
    -0.04652| 0.405465|  -1.4816|-0.526093|0.879249| 0.800778|  -1.4816|
   -0.893818|  -1.4816|  -1.4816| 0.800778|0.127833|-0.893818| 0.405465|
    0.276253|-0.257829| -0.04652|
T |-0.04652| 0.127833|0.405465|-0.893818|-0.893818| 0.127833|-0.257829|
   -0.526093| -0.04652|0.276253|-0.526093|-0.257829|-0.257829|-0.526093|
    0.519875|-0.893818| 0.62253|-0.526093| 0.276253|-0.526093|-0.893818|
     -1.4816|-0.257829|0.800778|-0.257829|-0.526093| 0.405465|-0.893818|
    -0.04652| 0.127833|0.405465|
```

Figure S3. The R2 versions of random position weight matrices constructed by randomly shuffling numbers within each column in the individual genuine matrices. **(A)** PWM1-R2 constructed from PWM1. **(B)** PWM2-R2 constructed from PWM2. **(C)** PWM3-R2 constructed from PWM3.
