# Supplementary material for: sRNAscanner: A Computational Tool for Intergenic Small RNA Detection in Bacterial Genomes
Source: PLoS One. 2010 Aug 5;5(8):e11970. doi: 10.1371/journal.pone.0011970 (PMC2916834; doi:10.1371/journal.pone.0011970)
Supplement: Figure S4 — R3 versions of random matrices. (0.03 MB PDF) [file pone.0011970.s009.pdf]

(A) PWM1-R3

```
A|-0.20067|0.167054| 0.969401| 0.820981|-0.788457|-0.788457|
C|-0.78845| -2.3979|-0.788457|  -2.3979|-0.788457|-0.788457|
G|0.435318|0.969401|  -2.3979|-0.200671|-0.200671|-0.788457|
T|0.167054| -2.3979|-0.200671|-0.200671| 0.820981| 0.969401|
```

(B) PWM2-R3

```
A|-0.788457|  1.21302|  -2.3979|-2.3979| 0.820981|  -2.3979|
C|-0.200671|-0.788457|  1.21302|1.31568|-0.788457| 0.167054|
G|-0.200671|  -2.3979|  -2.3979|-2.3979|-0.200671|-0.788457|
T| 0.646627|  -2.3979|-0.788457|-2.3979|-0.788457| 0.820981|
```

(C) PWM3-R3

```
A|-0.893818|-0.526093|-0.893818|-0.893818| 0.276253| -0.04652|-0.257829|
   0.405465| 0.127833|  -1.4816| -0.04652| -0.04652|-0.257829|-0.257829|
  -0.893818| 0.127833| 0.127833|-0.257829|-0.257829| 0.276253|-0.526093|
   0.879249|  -1.4816| 0.276253| 0.800778| -0.04652| 0.405465|  1.01983|
   0.127833| 0.127833| 0.276253|
C|  -1.4816| 0.405465|-0.526093| -0.04652| 0.276253| 0.127833| 0.127833|
  -0.257829|-0.893818| 0.127833| -0.04652|-0.526093|-0.893818|-0.257829|
   -0.04652| 0.127833|-0.257829|-0.526093|-0.526093| -0.04652| -0.04652|
   0.276253|  0.62253|-0.257829|  -1.4816| -0.04652|-0.257829|-0.893818|
   0.405465| 0.519875| 0.276253|
G|  1.01983|-0.257829| 0.800778| 0.405465|-0.04652|-0.257829|0.276253|
  -0.526093|  -1.4816|  0.71562| 0.405465|0.405465| 0.800778|0.405465|
   0.276253|-0.893818| 0.127833| 0.276253|-0.04652|-0.257829|0.405465|
   -3.09104| 0.127833| 0.405465|-0.257829|-0.04652|-0.893818| -1.4816|
  -0.893818|-0.526093|-0.257829|
T|-0.526093| 0.127833|-0.257829| 0.127833|-0.893818| 0.127833|-0.257829|
   0.127833| 0.800778|-0.526093|-0.526093| -0.04652|-0.526093| -0.04652|
   0.276253| 0.276253| -0.04652| 0.276253| 0.519875| -0.04652| -0.04652|
    -1.4816|-0.257829|-0.893818|-0.257829| 0.127833| 0.276253|-0.526093|
   -0.04652|-0.526093|-0.526093|
```

Figure S4. The R3 versions of random position weight matrices constructed by randomly shuffling columns within individual genuine matrices followed by randomly shuffling numbers within each column in these intermediate random matrices. **(A)** PWM1-R3 constructed from PWM1. **(B)** PWM2-R3 constructed from PWM2. **(C)** PWM3-R3 constructed from PWM3.
