# Supplementary material for: sRNAscanner: A Computational Tool for Intergenic Small RNA Detection in Bacterial Genomes
Source: PLoS One. 2010 Aug 5;5(8):e11970. doi: 10.1371/journal.pone.0011970 (PMC2916834; doi:10.1371/journal.pone.0011970)
Supplement: Figure S5 — TargetRNA-identified putative sRNA-mRNA interactions. (0.07 MB PDF) [file pone.0011970.s010.pdf]


Score: -89 *P* value: 9.81557e-05

```
sRNA3              41   GUUUUUCUUCGCA-UUC-UUUUUUAUC   65
                        |||||||:||:|| ||| |||||||||
STM2284 (glpA)     -2   CAAAAAGGAGUGUCAAGCAAAAAAUAG   -28
```

Score: -87 *P* value: 4.25713e-05

```
sRNA5              40   AGCUUUAUUGACUAUUCCUUUUU   62
                        |||: |||| |||||||||||||
STM1875 (yobA)     6    UCGGUAUAAGUGAUAAGGAAAAA   -17
```

Score: -87 *P* value: 0.000239255

```
sRNA7              3    GCGAUUAAGC-UGUUAAUAGCUUGAUUAUCUGUGACGUAUAGAGCGCU   49
                        || |:|||:| |::  |||: ||:||: ||| |||||||||:|  |||
STM0687 (ybfM)     18   CGGUGAUUUGCAUGCGUAUUCAAUUAGGAGAAACUGCAUAUUUAACGA   -30
```

Score: -86 *P* value: 0.000540291

```
sRNA11             242  AUAUUUUUCUCGGAUUAUAUUGAG   265
                        |||||||| ||:||||||||| ||
STM3984 (yigG)     2    UAUAAAAA-AGUCUAAUAUAAAUC   -21
```

Score: -78 *P* value: 0.000659353

```
sRNA16             26   GAAACGAGACAGUGAACAGUACCGUUUUCUUCGGUUUUUA-UGCCAUAGUA   75
                        |||| :|  ||||||||| ||  ||||||  || |::||  |:| |||::|
STM0225 (ynaI)     19   CUUUUUUAAGUCACUUGUAAUAACAAAAG--GCGAGGAAGGAUGCUAUUGU   -30
```

Score: -86 *P* value: 0.000760695

```
sRNA17             291  ACAAGGAC-GCCAGCCACUUUUUAGUUGUUUGAA   323
                        ||| |:|| ||| | | |||||| :|||||||||
STM0938 (ybjE)     4    UGUACUUGUCGGCCCGGGAAAAAAUAACAAACUU   -30
```

Score: -79 *P* value: 0.000144163

```
sRNA18          8    AUUUUCUGUAGCACUAUAAGUAUUAAUUUAUUGUUAUUUU    47
                     ||:|||| |  || :|| | ||:: ||| |||||||||||
STM1403 (sscB)  18   UAGAAGAAA--GUAGUAGU-AUGGAUAACUAACAAUAAAA    -19
```

Score: -89 *P* value: 4.07964e-05

```
sRNA19          28   AUAACUUCAUAUAUUGAAAUAAAUAAAUGC    57
                     ||| |||||  | :||||||||||||| ||
STM1426 (ribE)  2    UAUAGAAGUUGAGGACUUUAUUUAUUUUCG    -28
```

Score: -98 *P* value: 2.24624e-05

```
sRNA20          68   AUU-UUCGUUCAUUACCGCGCCUCUUGUC-UCCUGAAAAGUA    107
                     ||| |||||||||: | :| ||| ||||  | ||||||||||
STM2154 (mrp)   13   UAACAAGCAAGUAGGGCUGAGGAAAACAUCAAGACUUUUCAU    -29
```

Score: -250 *P* value: 4.66294e-15

```
sRNA21          226  AGGGGAGGGGAAGCAUUCAAUCGUAUGGUUGGCUUUAUGAAAAACGGUGU    275
                     ||||||||||||||||||||||||||||||||||||||||||||||||||
STM4316         20   UCCCCUCCCCUUCGUAAGUUAGCAUACCAACCGAAAUACUUUUUGCCACA    -30
```

Score: -74 *P* value: 0.000359706

```
sRNA22          13   CGCUGUCGUUUAAAAAAUAAAAGA    36
                     |:||::||:|||  ||||||||||
STM3773         -3   GUGAUGGCGAAUGCUUUAUUUUCU    -26
```

Score: -67 *P* value: 0.00299573

```
sRNA23          5    UUCGUGAAGGAAUGUUCUAA    24
                     ||| | || |||||||||||
STM0224 (rcsF)  -6   AAGGAGUUACUUACAAGAUU    -25
```

Score: -87 *P* value: 5.17551e-05

```
sRNA24            29   UUGUUUUU-A-UUAUUUCAUUUUUACAUUUUUGUUAGUG   65
                       |:|||||| | ||   |||||||||||||::| ||||:|
STM4370 (hemE)    20   AGCAAAAACUCAAGCCAGUAAAAAUGUAAGGA-AAUCGC   -18
```

Score: -84 *P* value: 0.000741386

```
sRNA25            91   AUCCGGCACCUGAGGAAUA   109
                       ||||| |||||||||||||
STM3766           -5   UAGGCAGUGGACUCCUUAU   -23
```

Score: -88 *P* value: 2.17524e-05

```
sRNA26             2   AUUUUUAAAAUUUUUUUGUCGUUAACUUUCUUGAUUUUGAAUUUUUAUGC   51
                       ||||||  | ||||||::| | ||| | :|||:||   |  |||||||||
STM1379 (orf48)   19   UAAAAAACUGAAAAAAGUA-CUAUUUAUGGAAUUA---CCGAAAAAUACG   -27
```

Score: -77 *P* value: 0.000272471

```
sRNA27            39   UUUAUCAUUUAUUUUAUCAUUA   60
                       ||||| | | ||||||||||||
STM4206           20   AAAUAUUUAUUAAAAUAGUAAU   -2
```

Score: -94 *P* value: 9.99577e-05

```
sRNA28            67   AAAAAAAUGUAAUUUAGCAUUUUUUGUUUUUUUUAAUGAAAUUUAUACCAAAAA   120
                       |||||||||| ||  |||  |||||:| |::||  || || ||| |||  ||||
STM0276           20   UUUUUUUACAGUAU-UCGGUAAAAAUAUAGGAA--UAAUUCAAA-AUGAAUUUU   -30
```

Score: -107 *P* value: 2.12733e-05

```
sRNA29           108   UUAUUAUCAAUUUAA-AUAGUUCUAUACAUUCUUAUUUUCUAUGGAAAAC   156
                       ||||||||||||| | |||||| |||| |:||: ||:    | :||||||
STM0335           16   AAUAAUAGUUAAAGUAUAUCAAUAUAUCUGAGG-UAG----UUUCUUUUG   -29
```

Score: -87 *P* value: 0.00057811

```
sRNA30             68   CUUUGUGCUAUUGUAAUCAUA-ACCAUUCUCAUUUACACUUUGUGCGGAAAU   118
                        |:| :|:|| || ||| |||| ||||||||| ||   | | |:|| ||| ||
STM3138            20   GGAUUAUGAAAAAAUUUGUAUAUGGUAAGAGGAA---UAAUAUACACCUGUA   -29
```

Score: -85 *P* value: 0.000315897

```
sRNA31             4    UAAAAUCAUAGGGUUAUUUAUUGUCGGC   31
                        |||||||||  |||:|||:||:|:||:|
STM4162 (thiF)     19   AUUUUAGUA--CCAGUAAGUAGCGGCUG   -7
```

Score: -95 *P* value: 5.20344e-05

```
sRNA32             94   CAGGGAGGU-CUCAUUCUUUAUUAUUGCUCCACUGUGUGGUGUGU   137
                        ||:|||::| |||||||||:     ||||||| | ||||:|||:|
STM3630 (dppA)     15   GUUCCUUUAUGAGUAAGAAG-----ACGAGGUUA-ACACUACAUA   -24
```

Score: -89 *P* value: 0.000195753

```
sRNA33             46   UAAUCACGCGAUUAAUUAAUACCACGCUAAUUGUUUCU   83
                        |||| |||| ||:  ||||||||| : ||||| |||||
STM1417 (ssaP)     10   AUUA-UGCG-UAGCGAAUUAUGGUAUCAUUAAAAAAGA   -26
```

Figure S5. Computationally-identified sRNA-mRNA base pairing predicted using the TargetRNA utility [41]. The set of 22 TargetRNA-supported sRNAscanner hits listed in Table 2 sRNAs and cognate target mRNAs are shown with corresponding predicted interacting positions. Target mRNA gene designations are shown in parentheses. The scores and P values shown are as determined by TargetRNA.
