# Supplementary material for: sRNAscanner: A Computational Tool for Intergenic Small RNA Detection in Bacterial Genomes
Source: PLoS One. 2010 Aug 5;5(8):e11970. doi: 10.1371/journal.pone.0011970 (PMC2916834; doi:10.1371/journal.pone.0011970)
Supplement: File S2 — Supplementary References. (0.03 MB PDF) [file pone.0011970.s012.pdf]

Supplementary References:

R1. Gudapaty S, Suzuki K, Wang X, Babitzke P, Romeo T (2001) Regulatory Interactions of Csr Components: the RNA Binding Protein CsrA Activates csrB Transcription in Escherichia coli. J Bacteriol 183: 6017-6027.

R2. Repoila F, Gottesman S (2003) Temperature Sensing by the *dsrA* promoter. J Bacteriol 185: 6609-6614.

R3. Opdyke AJ, Ju-Gyeong K, Storz G (2004) GadY, a Small-RNA Regulator of Acid Response Genes in *Escherichia coli*. J Bacteriol 186: 6698-6705.

R4. Urbanowski ML, Stauffer LT, Stauffer GV (2000) The gcvB gene encodes a small untranslated RNA involved in expression of the dipeptide and oligopeptide transport systems I *Escherichia coli*. Mol Microbiol 37: 856-868.

R5. Chen S, Zhang A, Blyn LB, Storz G (2004) MicC, a Second Small-RNA Regulator of Omp Protein Expression in *Escherichia coli*. J Bacteriol 186: 6689-6697.

R6. Ferrario M, Ernsting BR, Borst DW, Wiese II DE, Blumenthal RM, Matthews RG (1995) The Leucine-Responsive Regulatory Protein of *Escherichia coli* Negatively Regulates Transcription of *ompC* and micF and Positively Regulates Translation of *ompF*. J Bacteriol 177: 103-113.

R7. Altuvia S, Weinstein-Fischer D, Zhang A, Postow L, Storz G (1997) A Small, Stable RNA Induced by Oxidative Stress: Role as a Pleiotropic Regulator and Antimutator. Cell 90: 43-53.

R8. Lee Y, Ramamoorthy R, Chung-Ung P, Schmidt FJ (1989) Sites of Initiation and Pausing in the *Escherichia coli rnpB* (M1 RNA) Transcript. The Journal of Biol Chem 264: 5098-5103.

R9. Majdalani N, Chen S, Murrow J, St John K, Gottesman S (2001) Regulation of RpoS by a novel small RNA: the characterization of RprA. Mol Microbiol 39: 1382-1394.

R10. Antal M, Bordeau V, Douchin V, Felden B (2005) A Small Bacterial RNA Regulates a Putative ABC Transporter. The Journal of Biol Chem 280: 7901-7908.

R11. Wang AQ (1991) Regulation of biosynthesis in *Salmonella typhimurium*. Wei Sheng Wu Xue Bao. 31: 315-317.

R12. Peterson L, Downs DM (1996) Mutations in *apbC*(*mrp)* prevent function of the alternative pyrimidine biosynthetic pathway in *Salmonella typhimurium*. J Bacteriol 178: 5676-5682.

R13. Brown NL, Barrett SR, Camakaris J, Lee BT, Rouch DA (1995) Molecular genetics and transport analysis of the copper-resistance determinant (pco) from *Escherichia coli* plasmid pRJ1004. Mol Microbiol 17: 1153-1166.

R14. Mariscotti JF, Garcia-del Portillo F (2009) Genome expression analyses revealing the modulation of the *Salmonella* Rcs regulon by the attenuator IgaA. J Bacteriol 191: 1855-1867.

R15. Drew D, Sjostrand D, Nilsson J, Urbig T, Chin CN, de Gier JW, von Heijne G (2002) Rapid topology mapping of *Escherichia coli* inner-membrane proteins by prediction and PhoA/GFP fusion analysis. Proc Natl Acad Sci U.S.A. 99: 2690-2695.

R16. Bogomolnaya LM, Santiviago CA, Yang HJ, Baumler AJ, Andrews-Polymenis HL (2008) 'Form variation'of the O12 antigen is critical for persistence of *Salmonella* Typhimurium in the murine intestine. Mol Microbiol 70: 1105-1119.

R17. Frye J, Karlinsey JE, Felise HR, Marzolf B, Dowidar N, McClelland M, Hughes KT (2006) Identification of new flagellar genes of *Salmonella enterica* serovar Typhimurium. J Bacteriol 188: 2233-2243.

R18. Leonardi R, Roach PL (2004) Thiamine biosynthesis in *Escherichia coli:* in vitro reconstitution of the thiazole synthase activity. J Biol Chem 279: 17054-17062.

R19. Lewis JA, Horswill AR, Schwem BE, Escalante-Semerena JC (2004) The Tricarballylate Utilization (*tcuRABC*) Genes of *Salmonella enterica* Serovar Typhimurium LT2. J Bacteriol 186: 1629-1637.

R20. Kwon DH, Lu CD (2007) Polyamine effects on antibiotic susceptibility in bacteria. Antimicrob Agents Chemother 51: 2070-2077.

R21. Abouhamad WN, Manson MD (1994) The dipeptide permease of *Escherichia coli* closely resembles other bacterial transport systems and shows growth-phase-dependent expression. Mol Microbiol 14: 1077-1092.

R22. Sharma CM, Darfeuille F, Plantinga TH, Vogel J (2007) A small RNA regulates multiple ABC transporter mRNAs by targeting C/A rich elements inside and upstream of ribosome-binding sites. Genes Dev 21: 2804-2817.

R23. Harley CB, Reynolds RP (1987) Analysis of *E. coli* promoter sequences. Nucleic Acids Res 15: 2343-2361.

R24. Delcher AL, Bratke, KA, Powers EC, Salzberg SL (2007) Identifying bacterial genes and endosymbiont DNA with Glimmer. Bioinformatics 23: 673-679.
